# Myc Is a Metastasis Gene for Non-Small-Cell Lung Cancer

**DOI:** 10.1371/journal.pone.0006029

**Published:** 2009-06-24

**Authors:** Ulf R. Rapp, Christian Korn, Fatih Ceteci, Christiaan Karreman, Katharina Luetkenhaus, Valentina Serafin, Emanuele Zanucco, Inês Castro, Tamara Potapenko

**Affiliations:** 1 Department of Molecular Biology, Max-Planck-Institute of Biochemistry, München, Germany; 2 Department of Pathology, LMU, München, Germany; 3 Department of Biology, University of Konstanz, Konstanz, Germany; 4 Department of Microbiology, University of Würzburg, Würzburg, Germany; 5 University of Würzburg, MSZ, Würzburg, Germany; Health Canada, Canada

## Abstract

**Background:**

Metastasis is a process by which cancer cells learn to form satellite tumors in distant organs and represents the principle cause of death of patients with solid tumors. NSCLC is the most lethal human cancer due to its high rate of metastasis.

**Methodology/Principal Findings:**

Lack of a suitable animal model has so far hampered analysis of metastatic progression. We have examined c-MYC for its ability to induce metastasis in a C-RAF-driven mouse model for non-small-cell lung cancer. c-MYC alone induced frank tumor growth only after long latency at which time secondary mutations in K-Ras or LKB1 were detected reminiscent of human NSCLC. Combination with C-RAF led to immediate acceleration of tumor growth, conversion to papillary epithelial cells and angiogenic switch induction. Moreover, addition of c-MYC was sufficient to induce macrometastasis in liver and lymph nodes with short latency associated with lineage switch events. Thus we have generated the first conditional model for metastasis of NSCLC and identified a gene, c-MYC that is able to orchestrate all steps of this process.

**Conclusions/Significance:**

Potential markers for detection of metastasis were identified and validated for diagnosis of human biopsies. These markers may represent targets for future therapeutic intervention as they include genes such as Gata4 that are exclusively expressed during lung development.

## Introduction

Metastasis is a complex multistep process by which cells in the primary tumor become able to enter and leave the vasculature, settle at distant sites and eventually grow to macroscopic secondary tumors [1]. It is generally thought that metastatic conversion is a mutation driven process. A number of genes and gene products have been identified that positively or negatively affect the probability of established human tumor cell lines to metastasize [2], [3], [4]. Gene products include growth factor receptors and elements of their intracellular signal transduction cascades. Regulators of cell polarity represent a second class of genes that may promote metastasis [5], [6]. By use of a mouse lung cancer cell line, Lewis lung carcinoma (LLC), a third category of metastasis regulators has recently been described that highlighted the importance of inflammation as a driving force [7]. Moreover, candidate metastasis-inducing or -suppressing genes also include genes encoding micro RNAs that regulate sets of genes associated with proliferation, invasion or migration [8], [9].

While work with human tumor cell lines in mice has been enlightening, lack of knowledge about their repertoire of cancer genes as well as the inability to study the kinetics of spontaneous tumor progression in these models limit their usefulness. We have therefore established a mouse model for human non–small-cell lung cancer (NSCLC) in which premalignant adenoma is induced by a *RAF* transgene specifically expressed in lung alveolar type II epithelial cells [10]. NSCLC is the most frequent human lung cancer and the major cause of death from cancer in man. Generation of compound mice that carry in addition to *RAF* other transgenes that encode proteins modulating signaling pathways frequently altered in human NSCLC allowed us to unravel two key steps in the process of metastasis, induction of the angiogenic switch and progression to micrometastasis associated with reprogramming of intestinal selector genes [11]. β-catenin was identified as the critical switch inducer for both steps and also promoted invasive growth [11].

As 50% of human NSCLC carry mutations in genes that encode activators of the mitogenic cascade (RAS-RAF-MEK-ERK) [12] and this cascade is known to induce a target gene of ß-catenin, c-MYC [13], we decided to directly examine the ability of c-MYC to promote tumor progression in our RAF-driven murine lung cancer model. Moreover MYC was a candidate because it was known to affect reprogramming in tumors based on our earlier findings of RAF/MYC cooperation in the murine hematopoietic system [14], [15]. In humans, amplification and rearrangements of Myc genes were found in a fraction of NSCLC [16]. Several groups have begun to evaluate c-MYC in mouse models of NSCLC. Endogenous c-MYC is involved in non metastatic K-Ras-induced NSCLC as was shown by use of a dominant negative c-MYC mutant [17]. Inducible expression or function of transgenic c-MYC demonstrated cooperation with mutant K-Ras in tumor progression to various degrees [18], [19] but neither model gave rise to metastasis. Here we show that constitutive or inducible expression of c-MYC in addition to C-RAF in type II cells is sufficient to rapidly induce metastasis to liver and lymph nodes. Combining c-MYC and C-RAF transgenes caused appearance of a phenotypic switch from cuboidal to alveolar papillary/columnar epithelial cells (APECs) that are the most rapidly growing tumor cells and also predominate in liver metastasis. Moreover c-MYC induces production of VEGF by tumor cells leading to tumor vascularisation with unusual vessels that are mosaics containing PECAM-1 positive and PGP 9.5 positive endothelial cells. PGP 9.5 was previously known as a marker of immature neuroendocrine cells [20]. Metastatic conversion by c-MYC can be reconstructed in cell culture by using non-metastatic A-549 NSCLC cells and infection with a MYC transducing retrovirus.

## Results

### c-MYC cooperates with C-RAF in causing cancer related death

**Figure 1:**
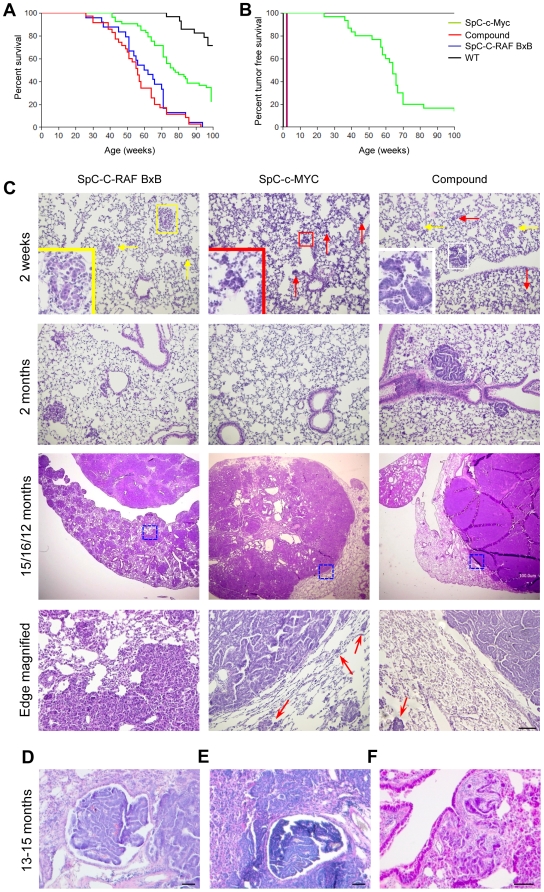
c-MYC cooperates with C-RAF in tumor growth. (A) Kaplan-Meier plot for SpC-C-RAF BxB (n = 24), SpC-c-MYC (n = 52), compound (n = 35) and wild-type (n = 28) littermate control mice. Log-rank analysis for percent survival for compound mice vs SpC-C-RAF BxB = p>0.05; SpC-C-RAF BxB vs SpC-c-MYC = p<0.0001; compound mice vs SpC-c-MYC = p<0.0001. n: number of animals. (B) Tumor incidence in SpC-C-RAF BxB (n = 51), SpC-c-MYC (n = 30), compound mice (n = 109) and wild-type littermate control mice (n = 58). Log-rank analysis for tumor free survival for SpC-C-RAF BxB vs SpC-c-MYC = p<0.0001; compound mice vs SpC-c-MYC = p<0.0001; wild type vs SpC-c-MYC = p<0.0001. n: number of animals. (C) Kinetics of tumor progression. Representative lung tumour sections stained with haematoxylin and eosin (H&E). The yellow box and arrows highlights cuboidal cell adenoma (inset) in SpC-C-RAF BxB mice; the red box and arrows indicates pleomorphic cells (inset) in SpC-c-MYC mice and the white boxes show a columnar cell adenoma (inset) in compound mice. For evaluation of invasiveness at the tumour-stroma interface, the boxed areas of the third panel are magnified in the bottom panel. Genotypes and ages of mice as indicated. Isolated small tumor cell clusters around the main tumor were indicated with red arrows. Scale bar: 100 µm. (D, E) H&E staining of paraffin embedded lung sections from compound (D) and SpC-c-MYC single transgenic (E) mice showing papillary tumors in bronchioles. Age of mice as indicated. (F) Desmoplastic progression of lung tumors from a compound mouse. Age of mice as indicated. Scale bar, 100 µm.

To test whether C-RAF and c-MYC cooperate in transformation of lung epithelial type II cells in vivo, SpC-C-RAF BxB mice were crossed with SpC-c-MYC transgenic mice. Observation for a period of ≥2 years demonstrated accelerated death in compound (SpC-C-RAF BxB/SpC-c-MYC) mice (Figure 1A) although the degree of acceleration was less than seen previously in the hematopoietic system with MYC/RAF retroviruses [21]. Nevertheless, compound mice had a median latency that significantly differed from single SpC-c-MYC transgenic mice by log-rank analysis. Histological examination at different time points revealed that single transgenic mice differed in tumor free survival. Whereas SpC-C-RAF BxB or compound mice were uniformly tumor positive at two weeks of age, SpC-c-MYC single transgenic mice developed tumors late and with incomplete penetrance indicating that c-MYC is not rate limiting for proliferation of type II cells and/or that responsive cells are eliminated by apoptosis (Figure 1B).

### Premalignant lesions in SpC-c-MYC single transgenic mice

Before onset of frank malignancy in SpC-c-MYC single transgenic mice clusters of pleomorphic cells are detected in the lungs that are potential sources for metastasis initiating cells (MICs) (Figure 1C). Intriguingly, pleomorphic clusters in lungs from long term survivors were often diminished or completely absent (data not shown). In order to characterise these potential premalignant lesions, expression of genes regulating proliferation and lung development was examined by immunostaining (Figure S1). As expected, strong nuclear c-MYC and expression of C-RAF at low endogenous levels was detected in these clusters (Figure S1). Pleomorphic cells proliferate as judged from PCNA staining and have a high rate of apoptosis which is presumably responsible for their lack of hyperplastic expansion (Figure S1 and Figure S2) which also sets these lesions apart from atypical adenomatous hyperplasia (AAH). Markers of type II pneumocytes are maintained in the c-MYC positive foci including Gata6 which drives TTF-1 expression [22]. In contrast Gata4 and its target gene mucin are not expressed (Figure S1). Of the downstream effectors of the Gata6-TTF-1 cascade only pro SP-C but not CCSP is expressed at low levels when compared to type II pneumocytes outside of the clusters demonstrating absence of bronchio alveolar stem cells (BASCs) (Figure S1) [23]. c-MYC expression in pleomorphic clusters is associated with induction of proliferation and apoptosis (Figure S1 and Figure S2). In fact by using a doxycycline (DOX) inducible c-MYC transgene, we observed massive apoptosis after one day induction and extensive loss of lung tissue four weeks after induction (Figure S3A–S3C). Prolongation of the induction period led to formation of pleomorphic clusters and isolated papillary lung tumors (Figure S3D–S3E).

We next looked at Aquaporin 5 expression that is shared between type I and type II pneumocytes [24] and found it to be maintained in pleomorphic clusters (Figure S1). To gain further insights into progenitor features of these cells, a series of markers of developmental pathways was examined. These include HNF-3ß, Sox9 [20], and factors that are notoriously involved in stem or progenitor cells such as Bmi-1 [25], ß-catenin and Id2 [20]. All of these genes were found to be expressed (Figure S1). Id2 has been described to mediate Myc signalling in conjunction with a retinoblastoma protein [26]. We conclude that cells in the high c-MYC expressing clusters have progenitor cell features but differ from BASCs.

### Rescue and accelerated growth of c-MYC transformed type II pneumocytes by C-RAF

**Figure 2:**
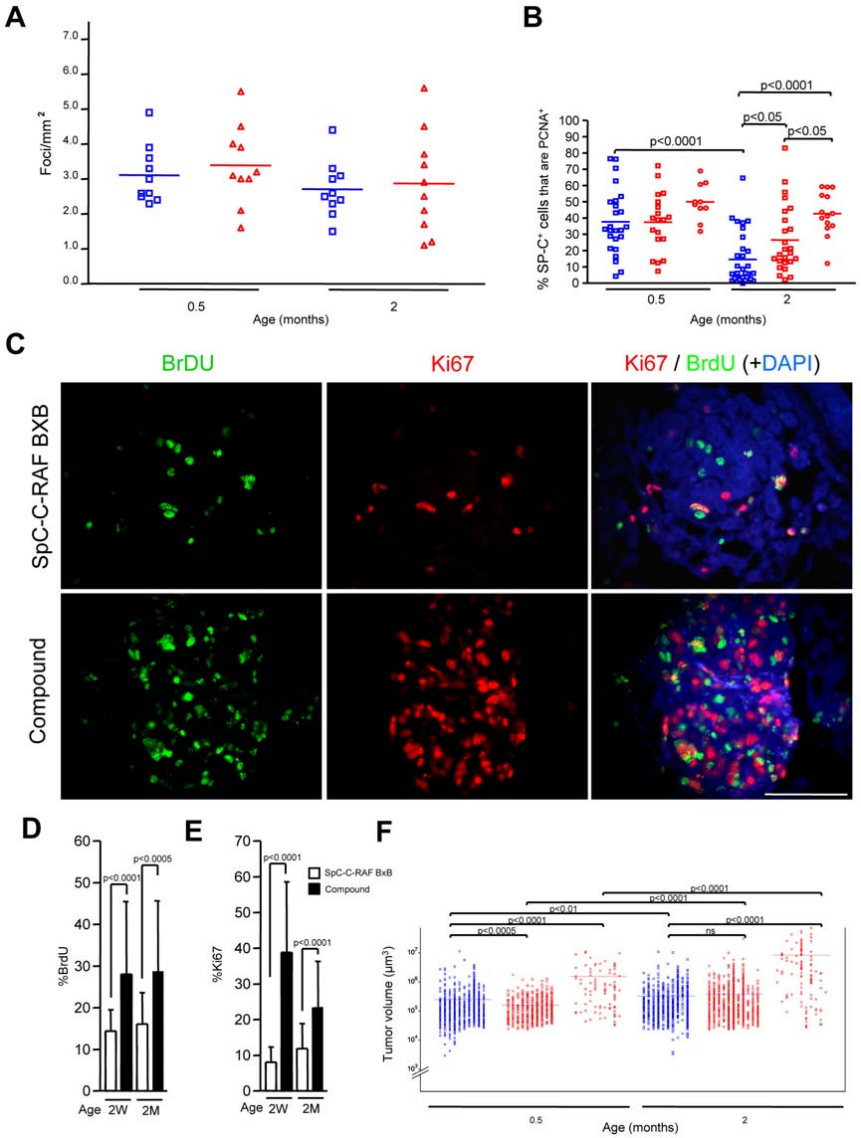
C-RAF and c-MYC cooperate in lung tumor acceleration but not in tumor incidence. (A) Tumor incidence in SpC-C-RAF BxB (blue open squares) and compound mice (red open triangle). Each symbol represents one lung. Values were normalised for lung size to compensate for age differences. There is no significant difference between the means. (B) Percentage of pro SP-C positive cells that are PCNA positive. Values represent SD of the mean. Genotypes and tumor categories are indicated as follows: SpC-C-RAF BxB cuboidal (blue open squares), compound cuboidal (red open squares) and compound columnar (red open circles). Each symbol represents one tumor. (C) Two weeks and two months old mice were injected daily with BrDU during one week and chased for one day. Paraffin embedded lung sections from SpC-C-RAF BxB single transgenic and compound mice were double stained for BrDU (green) and Ki67 (red) to identify cells in cycle. Dapi staining highlights lung tumors. Scale Bar: 100 µm. (D, E) Percentage of BrDU and Ki67 positive cells in tumors from both genotypes at different ages. 30 tumors from 3 mice were analysed per genotype. Values represent SD of the mean. (F) Tumor volume was computed by measuring tumor area assuming spherical shape of tumors. Symbols represent individual tumors. Each column represents one mouse. Genotypes and tumor categories are as described in B. All the tumors from each category were compared between genotypes. Values represent SD of the mean. Statistical differences between groups as indicated. ns: Not significant

In contrast to lungs of SpC-c-MYC single transgenic mice that only develop premalignant lesions, tumor nodules in lungs of compound or late SpC-c-MYC single transgenic mice were rapidly expanding and frequently contained macroscopic tumors with columnar cells that were absent in SpC-C-RAF BxB–driven adenomas (Figure 1C). Several lines of evidence indicate that columnar cells arise from cuboidal cells in compound mice. First, the tumor incidence at two weeks and two months of age is the same for SpC-C-RAF BxB single and SpC-C-RAF BxB/SpC-c-MYC compound mice (Figure 2A). Second, columnar cells mostly arise within cuboidal tumor foci (Figure S4A). Finally, columnar cells can be rapidly and frequently induced in vivo by DOX treatment of triple transgenic compound mice (SpC-C-RAF BxB/SpC-rtTA/tet-O-c-MYC) (Figure S4B and S4C). To test reversibility of c-MYC transgene expression DOX was removed one week after induction for a period of four weeks (Figure S4D). Histological analysis of the imaged lung tumor showed persistence of columnar cells in the center of one remaining tumor that otherwise displays a cuboidal phenotype (Figure S4D). Extension of the observation period after DOX removal to 10 weeks demonstrated elimination of columnar cells in tumors of all mice analyzed (data not shown). Comparison of tumor burden between continuously induced mice and mice that were taken off DOX for ten weeks after a four week induction period did not show significant differences (data not shown). These data suggest that a “shock from oncogene withdrawal” –effect [27] is responsible for the disappearance of APECs although reversibility of the MYC-induced cuboidal to columnar phenotypic switch is an alternative possibility.

Papillary tumors grow faster than cuboidal tumors of both genotypes. Consistently the fraction of PCNA positive, pro SP-C expressing cells was higher in papillary tumors (Figure 2B). Similar data were obtained by BrDU and Ki67 labeling (Figure 2C–2E). As expected from the proliferation data, tumor volumes increased most rapidly in papillary tumors of compound mice (Figure 2F). To further characterize columnar cells that are the hallmarks of advanced adenocarcinoma, we typed them for lineage and progenitor cell markers as in the case of the pre-malignant lesions in SpC-c-MYC single transgenic mice. Similar to pleomorphic clusters, columnar cells in early tumor foci from compound mice expressed progenitor cell markers (Figure S5A). A major difference however was the high level of C-RAF expression from the transgene which mediates rescue of cryptic c-MYC transformants from apoptosis (Figure S2A and S2B) leading to expansion of early tumor foci. In contrast, pleomorphic clusters in compound mice were similar to those in lungs of SpC-c-MYC single transgenic mice in terms of apoptosis rate (data not shown). Expression of progenitor cell markers in tumor foci of compound mice also differ in Id2 expression at late tumor stages when positive cells were very rare (Figure S5B). These data are consistent with reports of a tumor suppressor function of Id2 in the intestinal epithelium where it drives differentiation [28]. A third difference relates to homogeneity of transgene expression. Whereas c-MYC expression was uniform in all pleomorphic clusters, C-RAF and c-MYC transgene expression in columnar tumor foci was heterogeneous and decreased at late stages (Figure S5B) suggesting fluctuation of gene expression [29]. Finally, as in the case of pleomorphic clusters and in SpC-C-RAF BxB adenomas [30], BASCs were not detected at early or late stages in compound tumors (Figure S5A and S5B).

### c-MYC promotes tumor progression concomitant with angiogenesis in the absence of epithelial mesenchymal transition (EMT)

In compound mice tumor acceleration was evident histologically at all ages analyzed with frequent noninvasive macroscopic lung tumors which were absent in SpC-C-RAF BxB single transgenic mice (Figure 1C bottom panels). Macroscopic lung tumors generally were of the papillary type and classified as adenocarcinoma. In rare cases (2 in 50 cases for each genotype) we found papillary tumors occluding bronchioles and examples of desmoplasia in compound and SpC-c-MYC single transgenic mice, additional features that are reminiscent of human NSCLC pathology (Figure 1D–1F).

c-MYC has been reported to induce angiogenesis [31]. Significantly increased levels of blood and lymph vessels were detected in lung tumors of compound and SpC-c-MYC single transgenic mice (Figures S6A–C). The mechanism of angiogenic switch induction by c-MYC does not involve interference with E-cadherin expression in contrast to our earlier findings highlighting ß-catenin [11] as no nuclear ß-catenin was detected (Figure S5C). Moreover mast cells that are required for Myc-induced angiogenesis in pancreatic island tumors ([17] are absent from lung adenocarcinomas in mice of both genotypes (data not shown). Instead VEGF expression was detected in tumor cells of lung tumors from SpC-c-MYC single transgenic or compound mice (Figure S7A). Myc is also able to induce VEGF in human A-549 and mouse MLE-15 NSCLC cells in culture (Figure S7B). The combined data suggest that Myc induces angiogenesis by up-regulation of VEGF in NSCLC cells.

A striking observation was made when we explored potential neuroendocrine features of our NSCLC by staining with PGP 9.5. PGP 9.5 is an immature neuroendocrine marker and a candidate tumor marker for human NSCLC where it is expressed in tumor cells from >70% of stage II and IIIA tumors [32]. In our NSCLC model, the bulk of tumor cells are negative for this deubiquitinating enzyme at all stages independent of genotypes (data not shown). Instead, staining seems to mark vessels in the primary tumors of SpC-c-MYC single transgenic and compound mice (compare Figure S6D). In fact co-staining of tumor sections with PGP 9.5 and PECAM-1 showed colocalisation of these markers in the same vessels (Figure S6E). In order to examine whether PGP 9.5 is a marker of pathological angiogenesis, we also stained wild type lung sections where staining was found exclusively in large vessels but not in capillaries (Figure S6E) in line with a recent publication showing expression of PGP 9.5 in human carotid arteries [33]. Our findings are the first report regarding occurrence of such endothelial cells in the tumor vasculature.

The angiogenic switch was presumably not accompanied by typical epithelial mesenchymal transition (EMT) at the tumor edges as we did not detect cells doubly positive for pro SP-C and vimentin or pro SP-C and N-cadherin (Figure S8B). Moreover E-cadherin junctions of columnar cells in tumor remained intact (Figure S8A). Consistently we did not observe invasive fronts in papillary tumors (Figure 1C). Instead isolated groups of tumor cells were found in the vicinity of the primary tumors reminiscent of collective migration [34] (Figure 1C).

### Long latency tumors in SpC-c-MYC mice are positive for *K-Ras* or *LKB1* mutations

**Figure 3:**
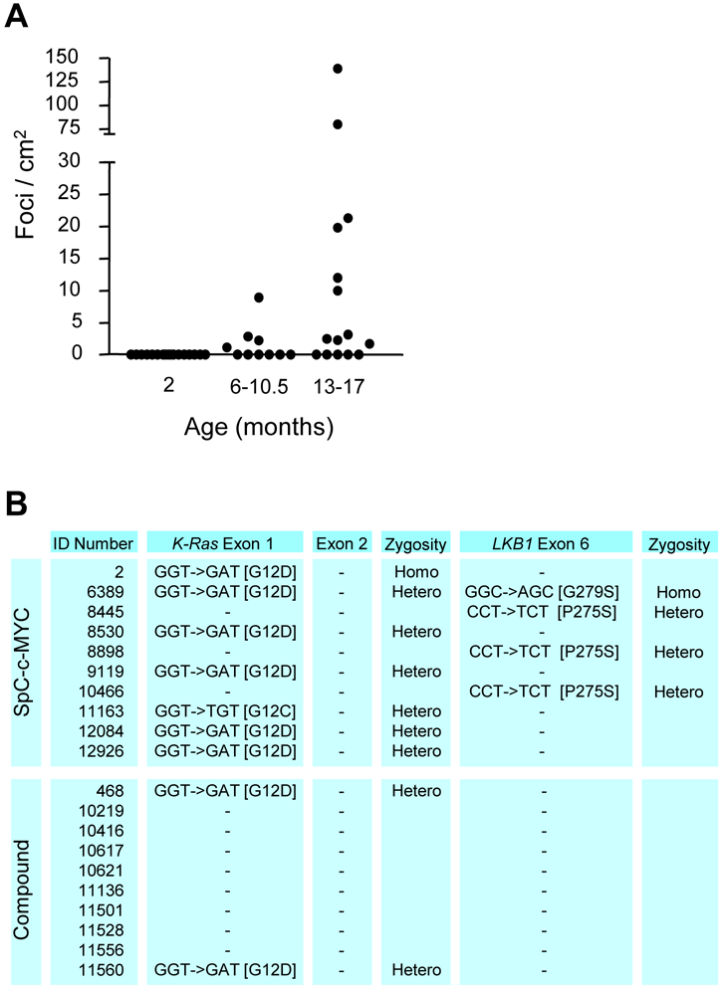
Long latency lung tumors from SpC-c-MYC single transgenic mice frequently carry *K-Ras* and *LKB1* mutations. (A) Tumor incidence per lung in SpC-c-MYC mice as a function of age. Animal numbers are 17 for 2 months, 10 for 6–10.5 months and 15 for 13–17 months. Each symbol represents an individual lung. Note that the ordinate is foci/cm^2^ as opposed to mm^2^ in Figure 2. (B) Occurrence of *K-Ras* and *LKB1* mutations in primary lung tumors. 10 randomly picked tumor positive mice were examined for a panel of candidate genes (*EGFR*, *K-Ras*, *B-RAF*, *C-RAF*, *p53*, *Pi3K*, *PTEN*, *Akt* and *LKB1*). Shown here are data for *K-Ras* and *LKB1*, the only genes of the panel in which mutations were found. Zygosity is as indicated. Individual mice are listed by ID numbers to facilitate comparison between tables.

As the kinetics of tumor development was delayed in SpC-c-MYC mice as compared to SpC-C-RAF BxB or compound mice and the number of late tumors per lung was lower (Figure 3A) we searched for secondary mutations. Toward this aim, a panel of genes (Text S1) that are frequently altered in human NSCLC was examined in a random collection of tumors from ten mice that led to the identification of frequent point mutations in *K-Ras* and *LKB1* genes (Figure 3B). Most *K-Ras* mutations were in exon 1 and were heterozygous. There was one case of an exon 2 mutation (Figure 5B) and in three cases no mutations were found in *K-Ras* or any other genes in the panel except *LKB1* (Figure 3B). In fact mutations in exon 6 of this tumor suppressor that is mutated in 34% of human NSCLC [35] were found in each of three *K-Ras* negative SpC-c-MYC lung tumors (Figure 3B). Only in one case were *LKB1*- and *K-Ras*- mutations found to coexist. In this example a different mutation of *LKB1* was observed which was present on both alleles (Figure 3B). In all other instances, *K-Ras* mutations or presence of oncogenic C-RAF were mutually exclusive with *LKB1* mutation (Figure 3B). This finding may suggest that K-Ras/C-RAF signaling in type II cells may normally lead to inactivation of this tumor suppressor. A similar relationship was found between *K-Ras* mutations and presence of oncogenic C-RAF, consistent with the mutually exclusive pattern of *RAF* and *RAS* mutations in human tumors [36] [37] (Figure 3B).

### Myc is sufficient to induce metastasis

**Figure 4:**
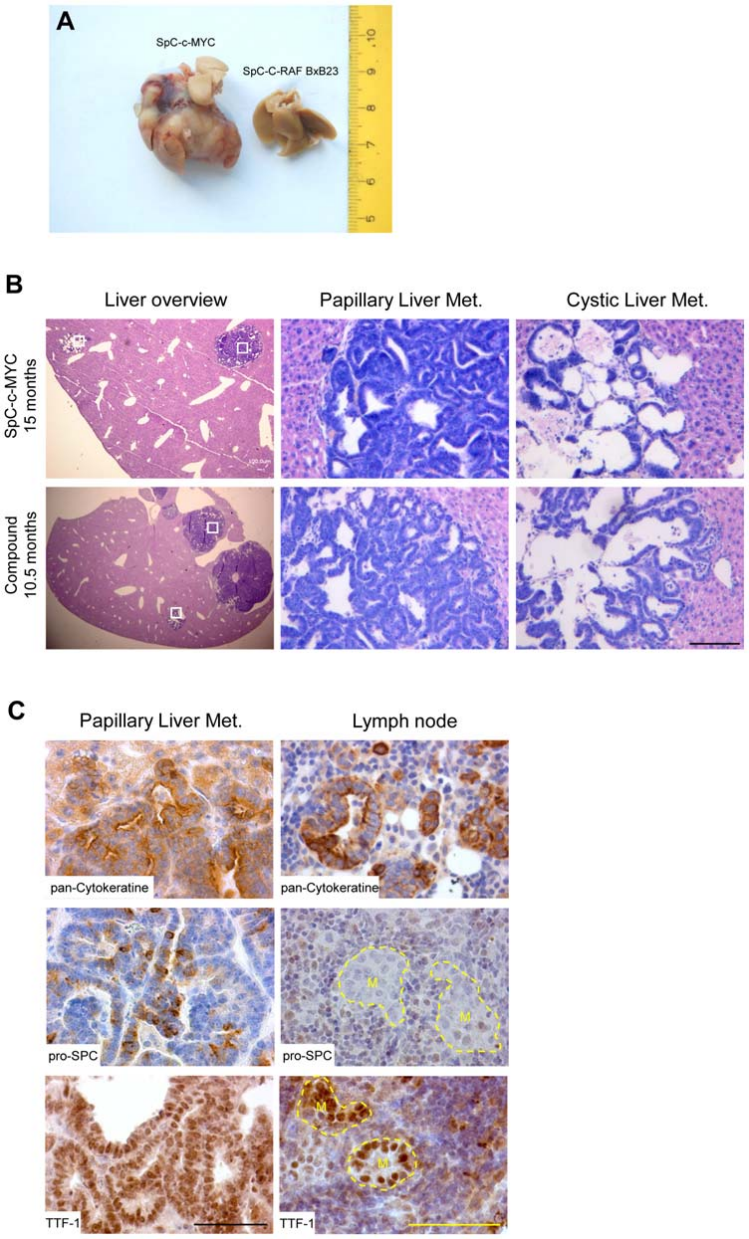
Macrometastases in liver and lymph nodes of SpC-c-MYC single transgenic and compound mice. (A) Inspection of liver metastasis (left) from a SpC-c-MYC single transgenic mouse. A normal liver (right) from a non-metastatic SpC-C-RAF BxB single transgenic mouse. (B) H&E staining of paraffin embedded liver sections from SpC-c-MYC and compound mice. Representative sections of papillary and cystic metastases are shown. Boxed areas are shown at higher magnification. Ages and genotypes as indicated. Scale bar: 100 µm. (C) Staining of liver and lymph node metastases from compound mice for markers as indicated. Metastases from SpC-c-MYC mice were indistinguishable when compared with compound mice for same markers. Circled areas in lymph nodes mark metastasis (M). Scale bar: 100 µm.

**Figure 5:**
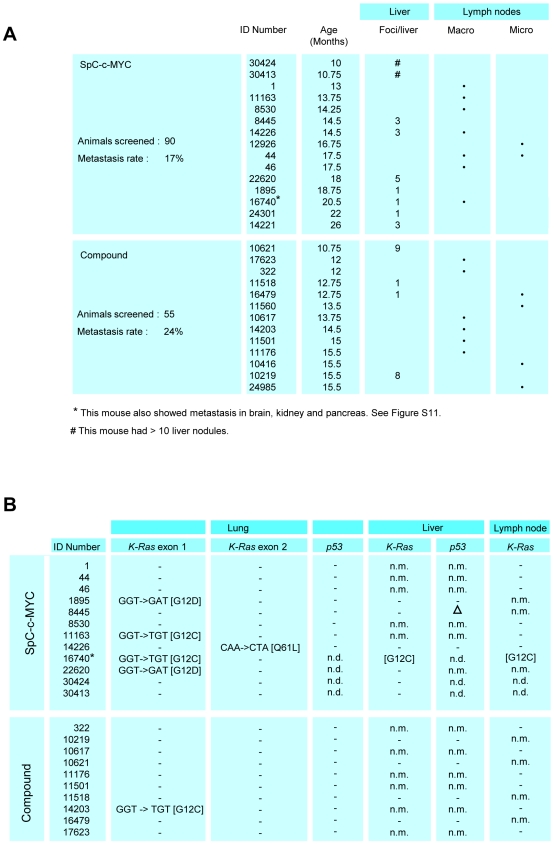
Summary of liver and lymph node metastases and their mutation status. (A) Summary of metastasis pattern for SpC-c-MYC single transgenic and compound mice. Animals are listed individually by ID number and grouped according to genotype. The median age of onset of metastases between the groups is significant (p = 0.03). (B) Absence of *K-Ras* mutation in macrometastasis of RAF/MYC and RAS/MYC lung tumors. n.m: no metastasis. n.d.: not done. Δ: Deletion. - : Negative.

As we have previously seen progression to micrometastasis in SpC-C-RAF BxB mice in which we provoked angiogenesis by ablation of E-cadherin, we examined mice for metastasis at different ages. Surprisingly multiple macroscopic liver- (Figure 4A and 4B) and lymph node- metastases (Figure 4C) were observed as early as ten months of age in compound (SpC-C-RAF BxB/SpC-c-MYC) and SpC-c-MYC single trasngenic animals. Compound mice developed metastasis significantly earlier, at higher incidence than SpC-c-MYC single transgenic mice (Figure 5A). Whereas in lymph nodes macro and micro metastasis were seen, only macro metastases were found in the liver. Lymph node and liver metastasis were independent events as were macro- and micro- metastasis suggesting that metastasis initiating cells (MICs) may differ for each category (Figure 5A). Besides SpC-c-MYC single transgenic mice, DOX inducible expression of c-MYC which rapidly gives rise to papillary lung tumors on the C-RAF BXB background (Figure S4) was examined for generation of metastasis. These mice developed lung tumors (Figure S9A) and liver metastasis (Figure S9C and 9D) made up of columnar cells. Consistent with the frequency of metastasis in constitutive mice, in a cohort of 10 inducible compound mice that were induced 11–12 months, one mouse developed macroscopic liver metastastasis and a second animal was positive for micro metastasis in a regional lymph node (Figure S9B). A cohort of age-matched control (uninduced) mice that were 14–17 months old did not show metastasis (data not shown). Similar to constitutive mice, sections from liver metastasis of inducible compound mice (Spc-C-RAF BxB/SpC-rtTA/tet-O-c-MYC) were positive for pro SP-C and pan-cytokeratin (Figure S9E and S9F). In contrast to constitutive mice, liver metastasis in inducible compound mice provoked a pronounced stromal response that separates tumor tissue from normal liver (Figure S9D). Notably, in this stroma-rich border area isolated clusters of tumor cells that were positive for pan-Cytokeratin or pro SP-C were present (Figure S9E and S9F) consistent with invasion by collective migration [34]. Upon examination of the mutational status of K-Ras, an exon 1mutation was found in primary lung tumors and corresponding liver metastasis (data not shown).

**Figure 6:**
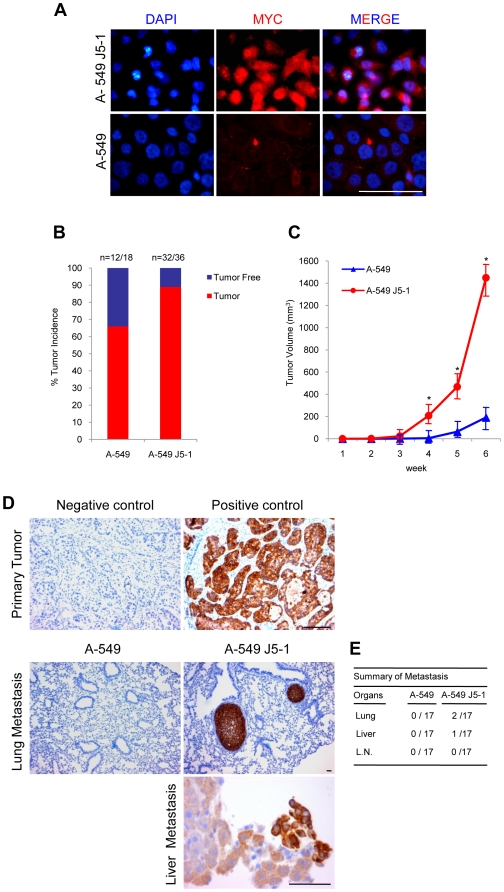
Increased tumor growth and rapid induction of metastatic switch in human A-549 cells by MYC. (A) Immunofluorescence staining of A-549 cells infected with chicken v-myc expressing J5 virus shows MYC (red) expression. Dapi marks nuclei. Scale bar: 50 µm. (B) Tumor incidence of control and MYC expressing A-549 cells six weeks after subcutaneous injection. Animal numbers (n) are as indicated. (C) Monitoring of tumor growth during six weeks of period. Tumors were measured weekly. MYC expressing cells gave rise to statistically larger tumors in Rag1^−/−^ mice. Values represent SD of the mean. *: p<0.05. n = 17 mice for each group. (D) Analysis of metastases. Paraffin embedded sections of lung, lymph node and liver tissues from transplanted mice were screened for metastasis by using cytokeratin-7 (CK-7) antibody. To demonstrate specificity of CK-7 expression in A-549 cells primary tumors were stained in the absence (negative control) or in the presence (positive control) of CK 7 antibody. For each organ at least 3 sections were stained and carefully screened at least by two persons. n = 17 mice for each group. Scale bar: 50 µm. (E) Incidence of Metastasis. L.N: Lymph node.

If acute expression of c-MYC is sufficient for metastatic conversion then introduction into non-metastatic NSCLC cells should generate metastatic clones. To test this possibility we employed human A-549 cells. A high c-MYC expressing A-549 cell clone (A-549 J5-1) (Figure 6A) was injected subcutaneously into immunodeficient Rag1^−/−^ mice. The incidence (Figure 6B) and rate of local tumor growth (Figure 6C) was increased by MYC expression to such a degree that animals had to be sacrificed after six weeks. In spite of this limited time span of the experiment, metastasis to lung and liver was observed at low frequency (Figure 6 D and 6E). Analysis of lung sections from A-549 J5-1 cells inoculated mice showed expression of chicken MYC in lung metastasis confirming the origin of cells (Figure S10). The combined data establish c-MYC as a strong metastasis inducing gene for NSCLC.

### Use of mutations in primary tumors of SpC-c-MYC single transgenic mice for lineage tracing of metastasis

The discovery of frequent *K-Ras* and *LKB1* mutations in lung tumors primarily of SpC-c-MYC single transgenic mice suggested that we might be able to use mutation data for lineage tracing. We therefore examined the DNA of liver and lymph node metastases for the mutations present in the primary tumors. Surprisingly in case of *K-Ras* mutations, four out of the five cases of c-MYC tumor derived metastases that could be examined in this way were negative for the mutation as was the one case of a compound mouse that had a *K-Ras* exon 1 mutation in the primary tumor and metastasis to lymph nodes (Figure 5B). However one out of the five cases of *K-Ras* positive c-MYC tumor derived metastasis was positive for mutant *K-Ras*. The mutation was identical to that in the lung tumor. This mouse had multiple organ metastases that included besides liver, kidney, pancreas and brain. Moreover, metastasis at different sites shared expression of lung markers. Figure S11 shows that scattered pro SP-C positive cells are present in distant metastasis in liver, pancreas, brain and kidney. Additionally in the kidney, Clara cell secretory protein (CCSP) positive tumor cells were detected (Figure S11). In the one case of a *LKB1*-mutant lung tumor that gave rise to liver metastasis, the metastasis scored negative for the mutation. The fact that mutations present in the primary lung tumors are mostly absent in their liver metastases strongly suggests that metastasis is an early event.

### Histopathology of metastasis from SpC-c-MYC single transgenic and compound mice

On histopathological examination metastases of both genotypes were indistinguishable except for their abundance (Figure 4A and Figure 5A). In both cases we find two types of metastasis in liver and lymph nodes, papillary and mixed cystic-papillary (Figure 4B). Commonly both forms coexist in the same target organ (Figure S12). Whether these two forms can interconvert or whether cysts are early precursor forms of mixed cystic-papillary metastasis is currently unknown. However the fact that we found one example of purely cystic liver metastasis which shows scattered pro SP-C positive cells in the lining epithelium (Figure S13) may argue that cysts may be a transitional state to development of frank malignancy.

**Figure 7:**
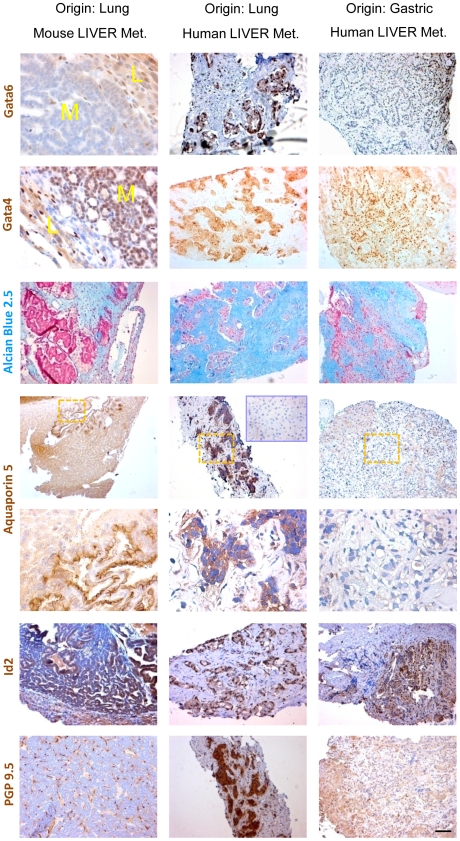
Human liver metastasis recapitulates the salient features of metastasis of mouse NSCLC. Paraffin embedded human liver biopsy sections were stained as indicated. L: liver, M: metastasis. Liver metastasis from lung and gastric cancers were compared. The lower of the two Aquaporin 5 panels is the higher magnification of the yellow boxes. Blue inset shows normal human liver that is negative for Aquaporin 5. Scale Bar: 100 µm.

C-RAF and c-MYC transgene expression was readily detected in liver metastasis (Figure S14). Whereas liver metastasis retained heterogeneous expression of pan-Cytokeratin, TTF-1 and pro SP-C, no pro SP-C expression was detected in lymph node metastasis indicating a more immature phenotype (Figure 4C). CCSP expression was not a uniform feature of liver metastasis of either genotype (data not shown). However there were positive cases with abundant expression (Figure S15A). This finding provided us with the opportunity to test for the presence of BASCs by searching for co-expression of pro SP-C and CCSP. Yet, attempts to detect double positive BASC cells in this metastasis were negative (Figure S15B) suggesting that CCSP positive cells may have been generated by multipotent cells without BASCs as intermediates. We therefore evaluated additional progenitor markers/lineage selectors for expression in tumor tissue of metastasis prone mice. Genes were selected based on their role during lung development and expression in emerging lung foci from compound mice. Candidates included Gata family members Gata6 and Gata4, Aquaporin 5, Id2 and PGP 9.5. The Gata6-Wnt pathway was recently shown to be required for epithelial stem cell development and airway regeneration [38]. Gata4 on the other hand is involved in maintenance of intestine in adult mice. Gata6 expression gradually decreased as a function of age in SpC-c-MYC single transgenic and compound mice in primary tumor (Figure S16A) and liver metastasis (Figure 7). Concomitantly strong nuclear Gata4 expression was detected in lung tumors (Figure S16A) as early as six months of age (Figure S17A) and their liver metastasis (Figure 7). In contrast, lung tumors of SpC-C-RAF BxB that are marked by stable Gata6 expression and remain Gata4 negative (Figure S16A). Mucin is a target gene of Gata4 in the intestine [39]. To establish functionality of ectopically expressed Gata4 in lung cells, we stained the primary lung tumors and liver metastases for mucin expression. Remarkably primary tumors and metastasis with columnar cells stained positive (Figure S16B and Figure 7) whereas tumors with cuboidal morphology were negative both in lungs of single SpC-C-RAF BxB transgenic and compound mice (Figure S16B). As an extension of these experiments we have examined whether Gata4 expression occurred at the expense of Gata6 and pro SP-C. As expected for a lineage switch process, large areas of mutually exclusive expression were found (Figure S17B). Another selector gene that marks intestinal lineages, Cdx2 was not expressed in primary tumors or metastasis of any of the genotypes (data not shown) highlighting Gata4 as a specific MYC target. Aquaporin 5 which was also detected in early expanding foci in the lung was strongly positive in liver metastasis (Figure 7). Finally PGP 9.5 which we have identified as a novel marker for tumor vasculature in mouse NSCLC was highly expressed in the vasculature of liver metastases (Figure 7). These markers may therefore be suitable for early detection of metastases of NSCLC.

### Human liver metastasis recapitulates the salient features of metastasis in the mouse

Mouse tumors do not always phenocopy their human correlates. It was therefore important to test whether changes observed during metastatic progression in the mouse also exist in human disease. Biopsy sections of liver metastasis from patients with NSCLC were first stained for the panel of markers found to be positive in mouse primary lung tumors and their corresponding liver metastasis. For control a primary tumor and the corresponding liver metastasis from stomach, a human tissue that normally expresses Gata4 was included.

As evident from Figure 7, there is a direct correspondence in Gata expression in the samples of both species. Three out of three liver metastasis of human NSCLC expressed Gata4 as illustrated in Figure 7 panel two. Expression of Gata4 is not as strongly confined to the nucleus as in the gastric liver metastasis (Figure 7 panel 3). The same pattern observed for Gata4 expression in human lung- to liver- metastasis is seen in the Alcian Blue 2.5 staining that identifies mucins emphasising the functionality of Gata4 (Figure 7). Gata6 expression in human lung- to liver- metastases shows a mutually exclusive pattern to Gata4 (Figure 7). We next stained the human liver metastases sections with Aquaporin 5. Aquaporin 5 staining is only present in mouse and human liver metastases that originate from lung. There was no staining of the human gastric- to liver- metastasis (Figure 7). This marker may therefore be suitable for early detection of metastases of NSCLC. The lung progenitor cell marker Id2 that was expressed in emerging foci from compound mice (Figure S5) was also present in human liver metastases independent of lung or gastric origin. Finally PGP 9.5 which we have identified as a novel marker for tumor vasculature in mouse NSCLC was highly expressed in human lung to liver metastases exclusively in tumor cells whereas tumor cells in human gastric to liver metastases were negative (Figure 7).

## Discussion

Here we have identified c-MYC as a metastasis inducer in a mouse model for NSCLC. Importantly these findings are not limited to mouse as the switch to metastasis was also observed in the human NSCLC cell line A-549 upon infection with MYC virus. We have used this model to identify novel markers for diagnosis of human lung to liver metastasis. The nature of these markers suggests that a lineage switch mechanism may be involved in progression to metastasis.

### c-MYC induces metastases

In our RAF-driven murine lung cancer model that does not progress spontaneously to an invasive or metastatic phenotype, we have shown previously that disruption of E-cadherin promotes progression to metastasis. Although accumulation of nuclear ß-catenin and up-regulation of c-MYC RNA occurred under these conditions, metastasis was limited to micrometastasis after a long latency period and did not include accumulation of nuclear c-MYC [11] and data not shown). In contrast, we show here for the first time that forced constitutive or inducible expression of nuclear c-MYC is sufficient to induce early macrometastasis of RAF-driven-NSCLC indicating that the level of c-MYC expression is a major determinant in this process. Metastasis was not described in the earliest report on oncogene transgenic mice utilizing c-MYC for induction of breast carcinoma [40] and pancreatic carcinoma [41]. Only recently a metastatic phenotype was reported for Ela-*myc* single and for MT-*tgf*α/Ela-*myc* double transgenic mice that gave rise to peritoneal and liver metastasis at an advanced stage [42]. More recently the role of c-MYC in mouse models of NSCLC was evaluated [18], [19], [43]. Even though c-MYC was shown to strongly cooperate with K-Ras in lung tumor progression, the role of c-MYC in this process could not be evaluated as neither model gave rise to metastasis. Why did these experimental strategies not reveal a metastasis function of c-MYC? There are major differences when compared to our model. First in the study that is most similar to ours as it used an inducible promoter and allowed for long term expression of c-MYC, a Clara cell specific- (CCSP) rather than alveolar type II cell specific-promoter (SpC) was used. This difference not only affects expression levels but examines c-MYC in different cells of origin of NSCLC (Clara cells instead of type II pneumocytes) and therefore may represent a different transformation pathway. The major difference of the second study relative to our work is the short term induction that for technical reasons was followed only for seven weeks. Furthermore in this study K-Ras was used instead of oncogenic C-RAF. The paucity of mutant K-Ras in liver metastases in our system may suggest that RAF is a stronger cooperation partner of MYC for metastatic progression perhaps because of differences in target cell specificity for transformation. In any case the model presented by us is highly robust and mimics the patterns observed for metastasis of human NSCLC.

### c-MYC requires cooperation with C-RAF, K-Ras or LKB1 in lung tumor progression

In our mouse NSCLC model, c-MYC alone was incapable of productive transformation let alone metastasis as its deleterious effects on cell survival blocked expansion of initiated cells. Only pockets of pleomorphic cells were present throughout the latency period. These clusters are potential targets for progression by secondary genetic events and therefore may have prognostic significance as premalignant lesions. In fact examination of long term survivors in the cohorts of SpC-c-MYC single transgenic mice showed lower abundance and in some cases lack of these premalignant lesions. Cells in these lesions differ from normal type II cells as they express high levels of c-MYC and its target Id2 [26] which is thought to mark multipotent lung progenitors during embryonic development [20]. Decreased levels of SpC expression in these cells are consistent with such a phenotype as the Myc effector Id2 is known to interfere with differentiation [44].

C-RAF cooperates with c-MYC in tumor progression by suppressing apoptosis. Alternatively c-MYC can be complemented by activation of K-Ras or LKB1. A second consequence of cooperation between these oncogenes is induction of a new alveolar papillary epithelial cell type, APEC. Induction of APECs is rapid and affects a large fraction of cells. These cells have the highest proliferation index in primary lung tumors and form the predominant cell type found in solid metastasis. Kinetic analysis of this switch strongly suggests epigenetic mechanisms as opposed to a mutation driven process as they arise rapidly and at high frequency. Marker gene expression indicates that the columnar cells are different from cells that were previously associated with lung cancer stem cells, BASC [23], or regeneration in the lung, i.e. variant clara cells [45] because they express Aquaporin 5 and are negative for CCSP. Moreover BASCs are not likely to be involved in metastasis because they are not detectable in late metastasis even when pro SP-C positive and CCSP positive single cells are abundant (Figure S14).

A similar phenotypic switch from cuboidal to columnar cells has been seen before upon deletion of p53 in SpC-C-RAF BxB transgenic mice [46] although at much lower frequency than in C-RAF/c-MYC compound mice. Since p53 has been reported to transcriptionally repress c-MYC [47] the simplest explanation for the earlier findings would be c-MYC up-regulation. Although the columnar cell switch is uniformly seen in compound and single transgenic mice only a fraction of them precedes to generate metastasis. Therefore we have to consider that additional events are required for the metastasis switch.

### Evidence for early generation of multipotent metastasis-initiating cells (MICs)

The earliest metastatic lesions in the liver of mice of any genotype were observed at ten months. At this time, the largest liver metastasis was larger than 5 grams and consisted of dozens of individual tumor foci. This observation provides the strongest argument for spread of MICs very early in the disease, perhaps as early as three to five months. Unfortunately detection of isolated seed cells in the liver was beyond our capacity because full examination would have required microscopic examination of thousands of sections. There are additional observations that support an early dissemination scenario. These include lack of secondary mutations in the majority of the metastasis, suggesting that premalignant pleomorphic clusters in SpC-c-MYC lungs might give rise to MICs. Moreover there were several examples with large metastasis in the liver and small tumor mass in the lung consistent with seeding of the liver at an early stage of tumor development. Finally the ability to convert A-549 human NSCLC cells to metastasising cells by forced MYC expression now enables us to study the sequence of events required for this switch in phenotype.

### Angiogenic switch induction by c-MYC

In addition to promoting an immature phenotype of tumor cells c-MYC accelerates growth of primary tumors by induction of angiogenesis. The ability of c-MYC to induce angiogenesis has been observed previously in other tumor models. In the pancreas a mechanism was delineated which involves mast cells [17]. In our mouse model for NSCLC we could not find any mast cells in the primary tumors or metastases (data not shown). On the other hand, MYC has been shown to induce angiogenesis in conjunction with HIF1 alpha by means of VEGF in lymphomas and epidermal lesions [31], [48]. In fact we did observe VEGF expression in our Myc expressing primary lung tumors and clones of Myc virus infected human A-549 and mouse MLE-15 NSCLC cells (Figure S7) extending these earlier findings to the lung.

In the course of examining tumors for expression of neuroendocrine function, we surprisingly detected high amounts of PGP 9.5 positive cells mainly in tumor vasculature. This pattern of expression was also found in the liver metastasis of mice as well as in normal large vessels. Interestingly PGP 9.5 was previously shown to be expressed by tumor cells in human NSCLC where it is used as a prognostic marker [32]. Based on the PGP 9.5 expression in normal human endothelial cells, the discrepancy between PGP 9.5 localisation might suggests that tumor cells can give rise to endothelial cells. In this case we would expect the PGP 9.5 positive endothelial cells of the tumor vasculature to also be positive for mutations present in the primary tumor. Future experiments will clarify this issue.

### Expression of novel markers in human lung to liver metastases

In order to evaluate relevance of our findings to human pathology, we have typed biopsy samples from human liver metastases for expression of our newly found markers of metastases. The pattern of Gata6, Gata4 and its target mucin as well as Id2 paralleled our findings in the mouse. Importantly Aquaporin 5 and PGP 9.5 expressions were unique to liver metastases from human NSCLC suggesting their usefulness for discrimination between tissues of origin. These findings also suggest that lineage switch events such as the Gata6-Gata4 exchange may drive progression to metastasis of NSCLC in mouse and man [14], [15]. Finally as Gata4 is dispensable for integrity of the adult lung, it may be an ideal target for development of future treatment regimens to treat lung cancer provided that therapy can be targeted to lung.

## Materials and Methods

### Animals

All animal studies were approved by the Bavarian State authorities for animal experimentation. Mice were housed under barrier condition in air-filtered, temperature-controlled units with a 12 h light/dark cycle with free access to food and water. Mice were monitored for signs of respiratory distress by inspection of their mobility and sacrificed before signs of extensive respiratory distress. All mice were of similar genetic background (≥90% C57Bl/6, ≤10% FVB/n) and maintained as heterozygotes. Compound mice were obtained by intercrossing heterozygotes. Other details of transgenic mice are described in Supporting Information (Text S1).

### RNA isolation and RT-PCR analysis

Semi quantitative RT-PCR was performed on total lung RNAs extracted from 6 weeks old mice using TRIZOL (Invitrogen) reagent as previously described ([11]. Oligos for human MYC sense: 5′- TTC CCC TAC CCT CTC AAC GAC AG – 3′and human MYC anti-sense: 5′- TCC TTA CTT TTC CTT ACG CAC AA- 3′were used for amplification of human c-MYC. Actin was used for loading control.

### ***In vivo*** bioluminescence imaging

Bioluminescence imaging of luciferase expression in compound mice (SpC-C-RAF BxB/SpC-rtTA/tet-O-c-MYC) was performed as described [49]. Briefly, mice were anesthetized with ketamine/rompun and subsequently received an i.p. injection of an aqueous solution of the substrate D-luciferin (125 mg/kg). The animals were then placed in a light-tight chamber and imaged with a CCCD camera. Images were acquired 20 minutes after luciferin administration. Signal intensity was quantified as the sum of all detected photon counts within the region of interest after subtraction of background luminescence.

### Immunofluorescence and Immunohistochemistry Microscopy

Animals were sacrificed and organs were fixed with 4% PBS buffered formalin. Histology was done on formalin-fixed, paraffin-embedded lung specimen. 6 µm-cut sections were deparaffinized, rehydrated in graded alcohols and haematoxylin-eosin stained. Details of staining protocols are given in Supporting Information (Text S1).

### Histopathology

Preparations of embedded tissues and quantitative assays for lung tumor development are described in Supporting Information (Text S1).

#### Statistical analysis

Tumour-free survival and comparisons of tumour numbers and tumour volume were analysed using GraphPad Prism4 software. Data are presented as mean±s.e.m. Student's *t* test (two-tailed) was used to compare two groups (*P*<0.05 was considered significant).

### Supplemental Data

Supplemental data include Supporting Information, 17 figures and one table.

## Supporting Information

Text S1 Supporting Material Methods and Supplementary figure legends
(0.16 MB DOC)

Figure S1 Premalignant lesions of SpC-c-MYC mice express type II pneumocyte- and progenitor- cell markers. Paraffin embedded lung sections were stained as indicated. Clusters of pleomorphic cells that represent premalignant lesions in SpC-c-MYC mice were highlighted with red circles. Scale bar: 50 µm.
(7.93 MB TIF)

Figure S2 Rescue of cryptic MYC transformants by co-expression of RAF. (A) Paraffin embedded lung sections from all genotypes were stained for active caspase 3 (brown) to detect apoptotic cells. Yellow circles identify neoplastic lesions. Jurkat cells treated or untreated with etoposide were used as positive and negative control, respectively. Haematoxylin (Blue) was used for counterstaining. Scale bar: 50 µm. (B) Quantitation of apoptotic cells for indicated genotypes. At least 40 randomly selected lesions from 3 mice per genotype were analysed for quantitation of apoptotic cells.
(2.28 MB TIF)

Figure S3 Conditional expression of c-MYC in lung alveolar type II cells induces tissue destruction. (A) Schematic diagram showing the generation of compound (SpC-rtTA/tet-O-c-MYC) mice conditionally expressing c-MYC in type II pneumocytes. Hs: Human. (B) Semi-quantitative RT-PCR showing inducible transgenic c-MYC (human) expression in lungs of compound mice after one week DOX administration. (C) Lung of a compound (SpC-rtTA/tet-O-c-MYC) mouse after four weeks induction shows severe tissue loss (white arrows) as evident from inspection of whole lung and the H&E stained section. Active caspase 3 staining identifies apoptotic cells (brown cells indicated by red arrows) in alveoli one day after doxycycline administration. Haematoxylin (blue) was used for counterstaining. (D) H&E staining of a lung section from four weeks-induced compound (SpC-rtTA/tet-O-c-MYC) mouse shows isolated pleomorphic cell clusters (yellow dash marking). (E) H&E staining of a lung section from 26 weeks-induced compound (SpC-rtTA/tet-O-c-MYC) mouse shows a lung adenocarcinoma with columnar cells. Scale bar: 100 µm.
(3.53 MB TIF)

Figure S4 Induction of phenotypic switch from cuboidal to alveolar papillary/columnar epithelial cells (APECs). (A) H&E staining of a mixed (cuboidal and columnar) lung tumor section from four months old compound (SpC-C-RAF BxB/SpC-c-MYC) mouse. (B) Schematic diagram showing the generation of triple transgenic compound (SpC-C-RAF BxB/SpC-rtTA/tetO-c-MYC) mice. (C) H&E staining of lung tumor sections from inducible (SpC-C-RAF BxB/SpC-rtTA/tetO-c-MYC) compound mice shows the kinetics of columnar cell appearance. D: day, W: week, M: month. Right hand panel is a magnification of the yellow box. Scale bar: 100 µm. (D) Six weeks old compound mice were imaged for in vivo luciferase expression following one week On DOX/4 weeks Off DOX schedule demonstrating inducibility. H&E staining of a lung tumor section of the On/Off DOX mouse. Inset highlights persistent papillary tumor area indicated by yellow box.
(3.75 MB TIF)

Figure S5 Immunostaining of emerging and late tumors of compound mice (SpC-C-RAF BxB/SpC-c-MYC) for lineage and progenitor cell markers. (A) Emerging tumors from 2 weeks-old mice were stained as indicated. (B) Late tumors from 10–16 months-old mice were stained as indicated. Markers are as indicated. In the case of fluorescence stainings colours correspond to the indicated proteins. Dapi (blue) illustrates nuclei. (C) β-catenin (green) staining of a lung tumor showing membrane localisation. Dapi (blue) illustrates nuclei.
(7.15 MB TIF)

Figure S6 Induction of angiogenic switch by c-MYC. (A) Immunostaining of lung tumor sections from control and metastatic animals of indicated genotypes for blood (Pecam 1) and lymph (Prox 1) vessels. Scale bar 100 µm. (B, C) Quantitation of vessel density. 5 mice per genotype were analysed. Values represent SD of mean. P values are as indicated. (D) PGP 9.5 immunostaining of lung tumor sections. Scale bar 100 µm. (E) Frozen lung sections from wild type (wt) and compound mice were stained for PGP 9.5 (red) and PECAM-1 (green) for co-expression. Dapi illustrates nuclei. Scale bar 50 µm.
(5.74 MB TIF)

Figure S7 VEGF induction by MYC in primary lung tumors and NSCLC cell lines. (A) Immunostaining of lung tumor sections from age-matched mice of the indicated genotypes for VEGF (brown). Haematoxylin (blue) was used for counterstaining. Scale bar: 100 µm. (B) Immunocytochemistry of human A-549 and mouse MLE-15 NSCLC cell lines for VEGF (green). Note increased VEGF expression in cells infected with Myc expressing retroviruses J5-1 and J2-11. One representative cell clone is shown for each virus. Dapi (blue) illustrates nuclei. Scale bar: 10 µm.
(3.77 MB TIF)

Figure S8 No evidence for EMT in tumor progression. (A) E-cadherin immunofluorescence staining (brown) of lung tumor section from a 12 months old compound mouse. Scale bar: 100 µm. (B) Paraffin embedded lung tumor sections from control and metastatic animals were stained for EMT markers as indicated. Tumor cells were marked by pro SP-C (green) staining. Dapi (blue) illustrates nuclei. Scale bar: 100 µm.
(3.53 MB TIF)

Figure S9 Inducible expression of c-MYC in SpC-C-RAF BxB lung tumors give rise to liver metastasis with APECs. (A) H&E staining of a lung section from a seven months induced compound mouse shows large lung tumors with columnar cell. Scale bar: 100 µm. (B) Pan-cytokeratin staining (brown) of a regional lymph node section from eleven months-induced compound mouse (SpC-C-RAF BxB/SpC-rtTA/tet-O-c-MYC) shows micrometastasis. (C) Inspection of a liver from a seven months induced compound (SpC-C-RAF BxB/SpC-rtTA/tet-O-c-MYC) mouse shows a tumor nodule in the liver (red circle). (D) H&E staining of the liver metastasis shown in C demonstrates papillary tumors with stroma (S). Scale bar: 100 µm. (E, F) Pan-cytokeratin (E) and pro SP-C immunostaining of the liver metastasis shown in C. Isolated pan-cytokeratin and pro SP-C positive tumor cells that were embedded in the stroma (S) were indicated by red arrows.
(6.17 MB TIF)

Figure S10 Staining of primary tumor and lung metastasis developing after subcutenous injection of A-549 J5-1 cells for chicken c-MYC.
(3.35 MB TIF)

Figure S11 Histopathology of metastasis to distant organs in old SpC-c-MYC mice. Multiple target organs are involved in case of a mutant K-Ras positive SpC-c-MYC lung tumor at age 20,5 months. Tissue sections were stained with indicated markers which identifies derivation of metastasis from lung adenocarcinoma. Haematoxylin (blue) was used for counterstaining.
(3.71 MB TIF)

Figure S12 Cystic and papillary forms coexist in late liver metastasis. (A) Macroscopic liver metastasis in an 18 months old SpC-c-MYC mouse. Note multiple solid tumor nodules (papillary, black arrows) and cystic lesion (yellow arrow). (B) H&E staining of liver metastasis confirm coexistence of both papillary and cystic (yellow circle) lesions in the same organ.
(2.83 MB TIF)

Figure S13 Cystic liver metastasis of a 13 months old SpC-c-MYC mouse. High magnification of red inset illustrates presence of pro SP-C positive (brown) cells (arrows) in the epithelial layer lining the cyst. Haematoxylin (blue) was used for counterstaining.
(1.35 MB TIF)

Figure S14 Expression of transgene markers in liver metastases. Immunofluorescence staining of papillary lung tumors and liver metastasis for expression of C-RAF (green) and c-MYC (red) transgenes.
(1.36 MB TIF)

Figure S15 No evidence for BASC in liver metastasis. (A) Paraffin embedded sections from 18 months liver metastasis were stained for CCSP and pro SP-C. Haematoxylin was used for counterstaining. Scale bar: 50 µm. (B) The same sections were subsequently examined for co-expression of CCSP (green) and pro SP-C (red) to search for BASCs. Dapi (blue) illustrates nuclei.
(3.65 MB TIF)

Figure S16 Gata6/Gata4 switch in SpC-c-MYC and compound mice. (A) Reciprocal expression of Gata4 and Gata6 in primary tumor of SpC-c-MYC and compound mice. Stainings as indicated. Note decrease in the level of Gata6 expression concomitant with heterogenous Gata4 expression in late stage lung tumors. Duodenum and embryonic lung sections were used for positive control for Gata4 and Gata6, respectively. (B) Alcian Blue 2.5 expression in lung tumors from indicated genotypes. Sections from 12 months old mice were stained with Alcian Blue for mucin secretion (blue stains, indicated by arrows).
(6.04 MB TIF)

Figure S17 Early and mutually exclusive expression of ectopic Gata4. (A) A representative lung section from a six-months-old compound (SpC-C-RAF BxB/SpC-c-MYC) mouse with an array of Gata4 expressing (brown) cells. (B) Immunohistochemistry for Gata4 and pro SP-C in serial sections of a lung tumor from a 18-monthd-old SpC-c-MYC single transgenic mouse illustrating mutually exclusive expression of both markers.
(2.63 MB TIF)
