# Supplementary material for: Myc Is a Metastasis Gene for Non-Small-Cell Lung Cancer
Source: PLoS One. 2009 Jun 24;4(6):e6029. doi: 10.1371/journal.pone.0006029 (PMC2696940; doi:10.1371/journal.pone.0006029)
Supplement: Text S1 — Supporting Material Methods and Supplementary figure legends (0.16 MB DOC) [file pone.0006029.s001.doc]

Supporting Information

**Myc is a Metastasis Gene for Non-Small-Cell Lung Cancer**

Ulf R. Rapp^1,2*^, Christian Korn^1,3^, Fatih Ceteci^1,2^, Christiaan Karreman^4^, Katharina Luetkenhaus^5^, Valentina Serafin^6^, Emanuele Zanucco^2^, Inês Castro^5^, Tamara Potapenko^1,6^

**Animals**

SpC-C-RAF BxB, SpC-c-MYC (gift from Roland Halter), SpC-rtTA (gift from Jeffrey A. Whitsett) and tet-O-c-MYC mice (gift from Thomas Wirth) have been previously described [1,2,3,4]. Genotype analysis of tail DNA was done by PCR at age three weeks.

**Cell culture and Transfections**

The A-549 cells were cultured in DMEM supplemented with 10% fetal calf serum (FCS). MLE-15 cells (a generous gift from Jeffrey A. Whitsett) were cultivated in HITES medium (RPMI 1640 medium supplemented with 10 nM hydrocortisone, 5 µg/ml insulin, 5 µg/ml human transferrin, 10 nM
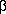
-estradiol, 5 µg/ml selenium, 2 mM L-glutamine, 10 mM HEPES, 100 units/ml penicillin, and 100 µg/ml streptomycin) supplemented with 2% fetal bovine serum.[[9]](http://www.sciencedirect.com/science?_ob=ArticleURL&_udi=B6WBK-4N43Y53-2&_user=616166&_rdoc=1&_fmt=&_orig=search&_sort=d&view=c&_acct=C000032339&_version=1&_urlVersion=0&_userid=616166&md5=61d3ea33d40bf88348fe4dfe647eae13#bbib9) Z. Borok, X. Li, V.F. Fernandes, B. Zhou, D.K. Ann and E.D. Crandall, Differential regulation of rat aquaporin-5 promoter/enhancer activities in lung and salivary epithelial cells, *J. Biol. Chem.* **275** (2000), pp. 26507–26514. [**Full Text** via CrossRef](http://www.sciencedirect.com/science?_ob=RedirectURL&_method=outwardLink&_partnerName=3&_targetURL=http%3A%2F%2Fdx.doi.org%2F10.1074%2Fjbc.M910007199&_acct=C000032339&_version=1&_userid=616166&md5=f3e1c16d501b93873347ca3774f2d58f) | [View Record in Scopus](http://www.sciencedirect.com/science?_ob=RedirectURL&_method=outwardLink&_partnerName=655&_targetURL=http%3A%2F%2Fwww.scopus.com%2Fscopus%2Finward%2Frecord.url%3Feid%3D2-s2.0-0034714337%26partnerID%3D10%26rel%3DR3.0.0%26md5%3Dd0c73c8a89a02a8e614c0c8fe0c558cf&_acct=C000032339&_version=1&_userid=616166&md5=211fa1ca305726a0956ac8daa65cfe89) | [Cited By in Scopus (19)](http://www.sciencedirect.com/science?_ob=RedirectURL&_method=outwardLink&_partnerName=656&_targetURL=http%3A%2F%2Fwww.scopus.com%2Fscopus%2Finward%2Fcitedby.url%3Feid%3D2-s2.0-0034714337%26partnerID%3D10%26rel%3DR3.0.0%26md5%3Dd0c73c8a89a02a8e614c0c8fe0c558cf&_acct=C000032339&_version=1&_userid=616166&md5=477c03ff7d519f7dbfefb7ddaf314f82) Cells were maintained in a humidified incubator at 37°C with 5% CO_2_ and harvested with 0.15% trypsin and 0.08% EDTA. For viral transfection, the J5 retrovirus, encoding a gag-myc fusion protein, was produced in the Phoenix amphotrophic packaging cell line. 10 µg of plasmid was transfected using Lipofectamine according to the manufacturer’s instructions (Invitrogen). Cells were given 24 hours to recuperate, after which the medium was exchanged. After overnight production, supernatant was harvested and used for infection of A-549 or MLE-15 cells in the presence of 8µg/µl polybrene. Three days post infection the cells were seeded in soft agar (0.3% agarose in DMEM with 10% foetal calf serum) in serial dilution. Single colonies that grew within 10 days to a size of over one mm were picked and transferred into 24-well plates.

**Histology**

Sacrification of mice and histological procedures were as described [5]**.** The numbers of tumor foci was calculated from whole lung sections with a cutoff of > 0.32 mm for tumor nodule diameter. Tumor volume (in mm^3^) was calculated assuming a spherical tumor shape.

**Immunohistochemistry and Immunofluoresence Microscopy**

Immunohistochemical analysis was performed as described [5]**.** Briefly, sections were deparaffinized and rehydrated. After 15 minutes incubation with 1-3 % H_2_O_2_ to quench endogenous peroxidase, sections were boiled in 10 mM sodium citrate buffer (pH: 6.0) for 6-30 minutes for antigen retrieval. Slides were blocked and incubated with relevant primary antibodies overnight at 4°C. Biotinylated secondary antibodies (Dako Cytomation) were applied to sections at 1:200-600 and incubated for 30-90 min at room temperature. ABC reagent (Vectastain Elite ABC Kit, Vector Labs) was applied to sections and developed in diaminobenzidine (DAB). Slides were then counterstained with haematoxylin. Primary antibodies against the following proteins were used: Aquaporin 5 (AQP-005, Alomone Labs), CCSP (sc-9772, Santa Cruz), PECAM-1 (clone MEC13.3, BD Pharmingen), C-RAF (sc-7267, Santa Cruz), pan-Cytokeratin (Z0622, Dako), Cytokeratin 7 (M7018, Dako), c-MYC (sc-764, Santa Cruz), chicken MYC (gift from Klaus Bister), Ki67 (NCL-Ki67-MM1, Novocastra), N-Cadherin (clone 3B9, Zymed), PCNA (PC10, BD Pharmingen), Pro SP-C (gift of Jeffrey A. Whitsett), Prox1 (102-PA30, Relia Tech), TTF-1 (M3575, Dako), Vimentin (C-20, Santa Cruz). ß-Catenin (06-734, upstate), Bmi-1 (sc-10745, Santa Cruz), Cleaved Caspase-3 (9664, Cell Signaling), E-cadherin (gp84, gift of R. Kemler), Gata4 (sc-1237, Santa Cruz), Gata6 (AF-1700, R&D Systems), HNF 3-β (sc-6554, Santa Cruz), PGP 9.5 (7863-0504, AbD Serotec) and Id2 (18-7398, Zymed). Double staining for pro SP-C and N-cadherin/Vimentin was done with paraffin embedded sections employing donkey anti-rabbit streptavidin Alexa Fluor ^488^ (Molecular Probes, Eugene, Oregon, USA) and donkey anti-mouse conjugated Cy-3 (Chemicon Int., USA) / donkey anti-goat Alexa Fluor ^555^ (Invitrogen) secondary antibodies, respectively. For PGP 9.5/PECAM-1 immunostaining, lungs were fixed in a cold paraformaldehyde in PBS for 4 h. After several rounds of washing, tissues were additionally incubated with 10% sucrose for 16 h before mounting in Tissue-Tek OCT. 10 µm-cut frozen lung sections were treated with primary antibodies after blocking and subsequently with secondary antibodies using Donkey anti-rat Alexa ^488^ and Donkey anti-rabbit Cy-3.

For immunocytochemistry, A-549 lung tumor cells were fixed in 4% PFA for 15 minutes and permeabilized with 0,2 % Triton X-100 for 10 minutes. After blocking with serum, primary antibodies were applied overnight at 4°C. After intensive washing with PBS, donkey anti-rabbit conjugated Cy-3 (1:300, Chemicon Int., USA) and goat anti-mouse conjugated Cy-3 (1:300, Jackson ImmunoResearch) secondary antibodies were employed for detection. Control sections were processed without primary antibody in all stainings.

The percentage of proliferating SP-C positive tumour cells (membrane associated Pro SP-C dots and nuclear PCNA signal) was obtained by dividing the number SP-C/PCNA double positive cells by the total number of SP-C positive cells. Fraction of apoptotic cells were determined using activated caspase 3 immunostaining and quantitated using 40 randomly selected areas from 3 mice per genotype. Paraffin-embedded Jurkat cells, untreated or etoposide-treated was used as positive and negative control for Caspase-3 staining (Asp175, Cell signaling). Intratumoral vessel density was evaluated as previously described [5]**.**

**Tumorigenicity in immunodeficient Rag1^-/-^ mice**

Six-week-old mice were injected subcutaneously with 1 X 10^6^ control and MYC expressing A-549 (A-549 J5-1) cells (dissolved in PBS) into the flanks of immunodeficient Rag1^-/-^ mice. Tumor size recorded weekly and tumor volume calculated using the formula 1/2 (length × width^2^). Mice were sacrificed when bleeding or opening occurred in the larger tumors.

**Isolation of tumor DNA and analysis of mutations**

Cells from individual small tumours were isolated from consecutive 10µm slides using a laser micro dissecting microscope (Leica ASLMD). For tumour recognition the slides were stained with haematoxylin. A minimal area of 50,000 µm^2^ was isolated and pooled for further processing. Large tumor DNA was isolated from two to three consecutive slides. Tissue was lightly counterstained with haematoxylin and tumor material was excised from the slide using a razor blade. DNA was isolated after solubilisation of the pooled material in 30 µl of lysis buffer (10mM TrisHCl, 1 mM EDTA, 1% w/v Tween 20, 100µg/ml proteinase K) o/n at 37º C. Proteinase K activity was destroyed by heating the samples to 95º C for twenty minutes. Probes were then diluted to 100µl and 1-2 µl of this was directly used for generation of chromosomal fragments using PCR. The following genes were checked for mutations: *p53* exon 7; *K-RAS* exon 1 and 2; *LKB1* exon 6, *PI3KCA* exon 10 and 21; *AKT* exon 1; *EGFR* exon 18, 19, 20 21; *PTEN* exon 5 and 8; *B-RAF* exon 18 and *C-RAF* exons 14 and 15. Primers for these genes are listed in Table S1.

References

1. Kerkhoff E, Fedorov LM, Siefken R, Walter AO, Papadopoulos T, et al. (2000) Lung-targeted expression of the c-Raf-1 kinase in transgenic mice exposes a novel oncogenic character of the wild-type protein. Cell Growth Differ 11: 185-190.

2. Ehrhardt A, Bartels T, Geick A, Klocke R, Paul D, et al. (2001) Development of pulmonary bronchiolo-alveolar adenocarcinomas in transgenic mice overexpressing murine c-myc and epidermal growth factor in alveolar type II pneumocytes. Br J Cancer 84: 813-818.

3. Marinkovic D, Marinkovic T, Mahr B, Hess J, Wirth T (2004) Reversible lymphomagenesis in conditionally c-MYC expressing mice. Int J Cancer 110: 336-342.

4. Perl AK, Tichelaar JW, Whitsett JA (2002) Conditional gene expression in the respiratory epithelium of the mouse. Transgenic Res 11: 21-29.

5. Ceteci F, Ceteci S, Karreman C, Kramer BW, Asan E, et al. (2007) Disruption of tumor cell adhesion promotes angiogenic switch and progression to micrometastasis in RAF-driven murine lung cancer. Cancer Cell 12: 145-159.

**Table S1. Primers for amplification of genomic DNA**

| Gene | Forward primer | Reverse primer |
| --- | --- | --- |
| *AKT* exon 1 | CCCATGCAGCTCCTTATGTC | AACCCGCATGGCTAAGACAC |
| *B-RAF* exon18 | TTCCTTTACTTACTGCACCTCAG | GCATGTGCAATTATGCCTGGC |
| *C-RAF* exon 14 | GAAGGAGGCCAGAAGCACTG | TTTTGCACCCTACTCTGGCC |
| *C-RAF* exon 15 | TGTCTCTGGAGGTCATTCCC | AAGTAGCCCCTCACTGTGTC |
| *EGFR* exon 18 | TGGTAGCATCTCAGGTCTGC | TGTCTCCAGGAAGCCTAGTG |
| *EGFR* exon 19 | CCAGCTCACAAGGCAACATG | CCCACGTCCCTATAAGCAGA |
| *EGFR* exon 20 | AAGGGATATGCGTGCCTCTC | GGGTACTTCAGTGGACAGAC |
| *EGFR* exon 21 | ACCCTGTGTTCAGGTGCATG | CTGGGCTGTCAGGAAAATGC |
| *K-RAS* exon 1 | ATGACTGAGTATAAACTTGT | TCGTACTCATCCTCAAAGTG |
| *K-RAS* exon 2 | TACAGGAAACAAGTAGTAATTGATGGAGAA | ATAATGGTGAATATCTTCAAATGATTTAGT |
| *LKB1* exon 6 | ACCCTGTAGCGGGGGG | CCTCCCATCCGGACAA |
| *p5*3 exon 7 | CCACAGGTCTCCCCAAGG | TGGCAAGTGGCTCCTGAC |
| *PIK3CA* exon 10 | CCAAGGAAATCATGGCAGAG | TCCAGCCTTGAGAGCCTCAC |
| *PIK3CA* exon 21 | CCGAAAGACTCTAGCCTTGG | ACTGCCATGCAGTGGAGAAG |
| *PTEN* exon 5 | GCAACAGTTGCACAGTATCCT | AATAAAACACACCCTCCCACC |
| *PTEN* exon 8 | CCACAAGGTGTTTGCCTTCAC | TCCACAAAGAGGGAGGAAGG |

**Figure S1. Premalignant lesions of SpC-c-MYC mice express type II pneumocyte- and progenitor- cell markers.** Paraffin embedded lung sections were stained as indicated. Clusters of pleomorphic cells that represent premalignant lesions in SpC-c-MYC mice were highlighted with red circles. Scale bar: 50 µm.

**Figure S2. Rescue of cryptic MYC transformants by co-expression of RAF.**

**(A)** Paraffin embedded lung sections from all genotypes were stained for active caspase 3 (brown) to detect apoptotic cells. Yellow circles identify neoplastic lesions. Jurkat cells treated or untreated with etoposide were used as positive and negative control, respectively. Haematoxylin (Blue) was used for counterstaining. Scale bar: 50 µm.

**(B)** Quantitation of apoptotic cells for indicated genotypes. At least 40 randomly selected lesions from 3 mice per genotype were analysed for quantitation of apoptotic cells.

**Figure S3. Conditional expression of c-MYC in lung alveolar type II cells induces tissue destruction.**

**(A)** Schematic diagram showing the generation of compound (SpC-rtTA/tet-O-c-MYC) mice conditionally expressing c-MYC in type II pneumocytes. Hs: Human.

**(B)** Semi-quantitative RT-PCR showing inducible transgenic c-MYC (human) expression in lungs of compound mice after one week DOX administration.

**(C)** Lung of a compound (SpC-rtTA/tet-O-c-MYC) mouse after four weeks induction shows severe tissue loss (white arrows) as evident from inspection of whole lung and the H&E stained section. Active caspase 3 staining identifies apoptotic cells (brown cells indicated by red arrows) in alveoli one day after doxycycline administration. Haematoxylin (blue) was used for counterstaining.

**(D)** H&E staining of a lung section from four weeks-induced compound (SpC-rtTA/tet-O-c-MYC) mouse shows isolated pleomorphic cell clusters (yellow dash marking).

**(E)** H&E staining of a lung section from 26 weeks-induced compound (SpC-rtTA/tet-O-c-MYC) mouse shows a lung adenocarcinoma with columnar cells. Scale bar: 100 µm.

**Figure S4. Induction of phenotypic switch from cuboidal to alveolar papillary/columnar epithelial cells (APECs).**

**(A)** H&E staining of a mixed (cuboidal and columnar) lung tumor section from four months old compound (SpC-C-RAF BxB/SpC-c-MYC) mouse.

**(B)** Schematic diagram showing the generation of triple transgenic compound (SpC-C-RAF BxB/SpC-rtTA/tetO-c-MYC) mice.

**(C)** H&E staining of lung tumor sections from inducible (SpC-C-RAF BxB /SpC-rtTA/tetO-c-MYC) compound mice shows the kinetics of columnar cell appearance. D: day, W: week, M: month. Right hand panel is a magnification of the yellow box. Scale bar: 100 µm.

**(D)** Six weeks old compound mice were imaged for in vivo luciferase expression following one week On DOX / 4 weeks Off DOX schedule demonstrating inducibility. H&E staining of a lung tumor section of the On/Off DOX mouse. Inset highlights persistent papillary tumor area indicated by yellow box.

**Figure S5. Immunostaining of emerging and late tumors of compound mice (SpC-C-RAF BxB/SpC-c-MYC) for lineage and progenitor cell markers.**

**(A)** Emerging tumors from 2 weeks-old mice were stained as indicated.

**(B)** Late tumors from 10-16 months-old mice were stained as indicated. Markers are as indicated. In the case of fluorescence stainings colours correspond to the indicated proteins. Dapi (blue) illustrates nuclei.

**(C)** ß-catenin (green) staining of a lung tumor showing membrane localisation. Dapi (blue) illustrates nuclei.

**Figure S6. Induction of angiogenic switch by c-MYC.**

**(A)** Immunostaining of lung tumor sections from control and metastatic animals of indicated genotypes for blood (Pecam 1) and lymph (Prox 1) vessels. Scale bar 100 µm.

**(B, C)** Quantitation of vessel density. 5 mice per genotype were analysed. Values represent SD of mean. P values are as indicated.

**(D)** PGP 9.5 immunostaining of lung tumor sections. Scale bar 100 µm.

**(E)** Frozen lung sections from wild type (wt) and compound mice were stained for PGP 9.5 (red) and PECAM-1 (green) for co-expression. Dapi illustrates nuclei. Scale bar 50 µm.

**Figure S7. VEGF induction by MYC in primary lung tumors and NSCLC cell lines.**

**(A)** Immunostaining of lung tumor sections from age-matched mice of the indicated genotypes for VEGF (brown). Haematoxylin (blue) was used for counterstaining. Scale bar: 100 µm.

**(B)** Immunocytochemistry of human A-549 and mouse MLE-15 NSCLC cell lines for VEGF (green). Note increased VEGF expression in cells infected with Myc expressing retroviruses J5-1 and J2-11. One representative cell clone is shown for each virus. Dapi (blue) illustrates nuclei. Scale bar: 10 µm.

**Figure S8. No evidence for EMT in tumor progression.**

**(A)** E-cadherin immunofluorescence staining (brown) of lung tumor section from a 12 months old compound mouse. Scale bar: 100 µm.

**(B)** Paraffin embedded lung tumor sections from control and metastatic animals were stained for EMT markers as indicated. Tumor cells were marked by pro SP-C (green) staining. Dapi (blue) illustrates nuclei. Scale bar: 100 µm.

**Figure S9. Inducible expression of c-MYC in SpC-C-RAF BxB lung tumors give rise to liver metastasis with APECs.**

**(A)** H&E staining of a lung section from a seven months induced compound mouse shows large lung tumors with columnar cell. Scale bar: 100 µm.

**(B)** Pan-cytokeratin staining (brown) of a regional lymph node section from eleven months-induced compound mouse (SpC-C-RAF BxB / SpC-rtTA / tet-O-c-MYC) shows micrometastasis.

**(C)** Inspection of a liver from a seven months induced compound (SpC-C-RAF BxB / SpC-rtTA / tet-O-c-MYC) mouse shows a tumor nodule in the liver (red circle).

**(D)** H&E staining of the liver metastasis shown in C demonstrates papillary tumors with stroma (S). Scale bar: 100 µm.

**(E, F)** Pan-cytokeratin (E) and pro SP-C immunostaining of the liver metastasis shown in C. Isolated pan-cytokeratin and pro SP-C positive tumor cells that were embedded in the stroma (S) were indicated by red arrows.

**Figure S10. Staining of primary tumor and lung metastasis developing after subcutenous injection of A-549 J5-1 cells for chicken c-MYC.**

**Figure S11. Histopathology of metastasis to distant organs in old SpC-c-MYC mice.**

Multiple target organs are involved in case of a mutant *K-Ras* positive SpC-c-MYC lung tumor at age 20,5 months. Tissue sections were stained with indicated markers which identifies derivation of metastasis from lung adenocarcinoma. Haematoxylin (blue) was used for counterstaining.

**Figure S12. Cystic and papillary forms coexist in late liver metastasis.**

**(A)** Macroscopic liver metastasis in an 18 months old SpC-c-MYC mouse. Note multiple solid tumor nodules (papillary, black arrows) and cystic lesion (yellow arrow).

**(B)** H&E staining of liver metastasis confirm coexistence of both papillary and cystic (yellow circle) lesions in the same organ.

**Figure S13. Cystic liver metastasis of a 13 months old SpC-c-MYC mouse.** High magnification of red inset illustrates presence of pro SP-C positive (brown) cells (arrows) in the epithelial layer lining the cyst. Haematoxylin (blue) was used for counterstaining.

**Figure S14. Expression of transgene markers in liver metastases.**

Immunofluorescence staining of papillary lung tumors and liver metastasis for expression of C-RAF (green) and c-MYC (red) transgenes.

**Figure S15. No evidence for BASC in liver metastasis.**

**(A)** Paraffin embedded sections from 18 months liver metastasis were stained for CCSP and pro SP-C. Haematoxylin was used for counterstaining. Scale bar: 50 µm.

**(B)** The same sections were subsequently examined for co-expression of CCSP (green) and pro SP-C (red) to search for BASCs. Dapi (blue) illustrates nuclei.

**Figure S16. Gata6/Gata4 switch in SpC-c-MYC and compound mice.**

**(A)** Reciprocal expression of Gata4 and Gata6 in primary tumor of SpC-c-MYC and compound mice. Stainings as indicated. Note decrease in the level of Gata6 expression concomitant with heterogenous Gata4 expression in late stage lung tumors. Duodenum and embryonic lung sections were used for positive control for Gata4 and Gata6, respectively.

**(B)** Alcian Blue 2.5 expression in lung tumors from indicated genotypes. Sections from 12 months old mice were stained with Alcian Blue for mucin secretion (blue stains, indicated by arrows).

**Figure S17. Early and mutually exclusive expression of ectopic Gata4.**

**(A)** A representative lung section from a six-months-old compound (SpC-C-RAF BxB/SpC-c-MYC) mouse with an array of Gata4 expressing (brown) cells.

**(B)** Immunohistochemistry for Gata4 and pro SP-C in serial sections of a lung tumor from a 18-monthd-old SpC-c-MYC single transgenic mouse illustrating mutually exclusive expression of both markers.
