# Supplementary figures and images for: Myc Is a Metastasis Gene for Non-Small-Cell Lung Cancer
Source: PLoS One. 2009 Jun 24;4(6):e6029. doi: 10.1371/journal.pone.0006029 (PMC2696940; doi:10.1371/journal.pone.0006029)

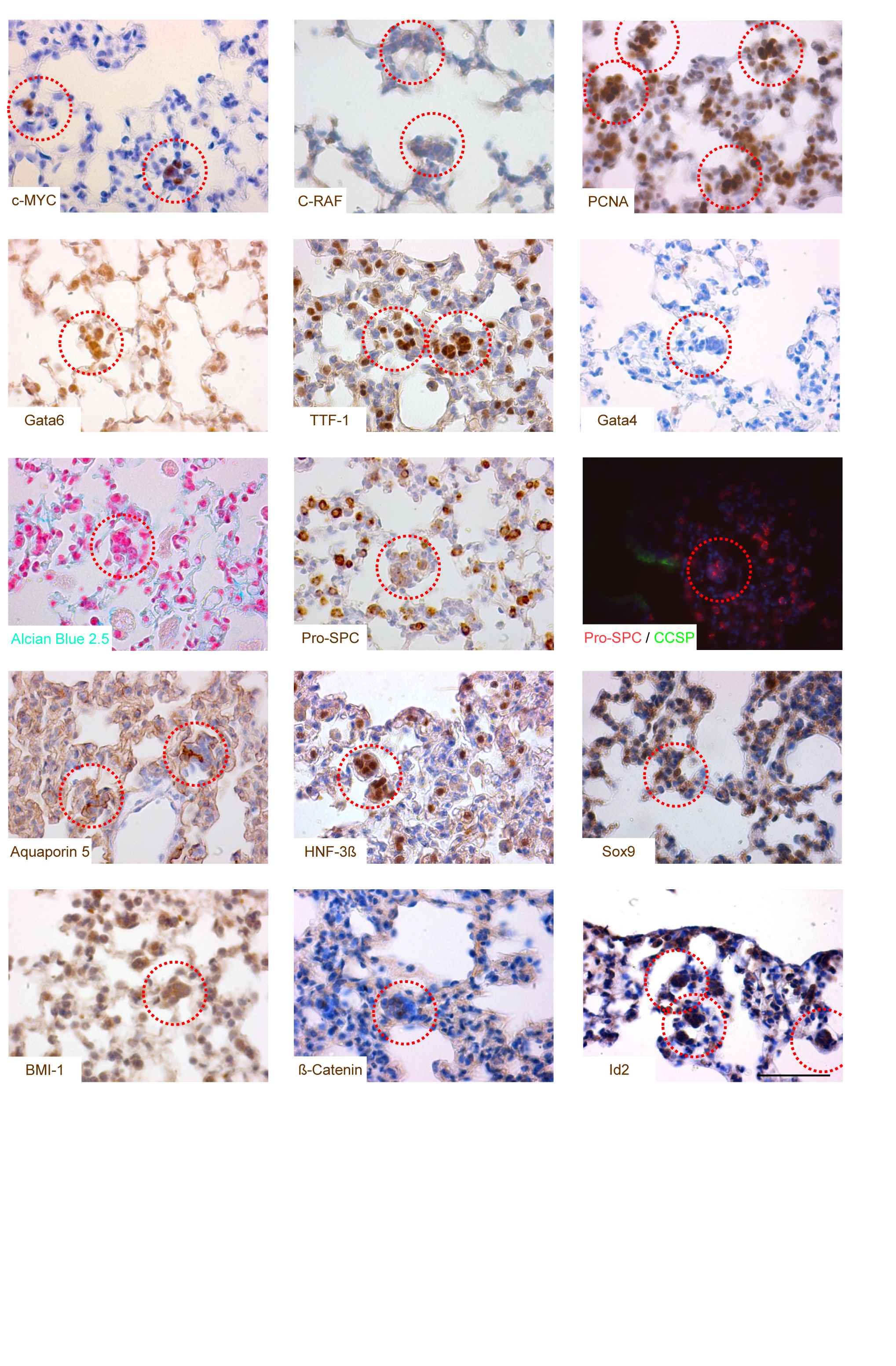

Supplement: Figure S1 — Premalignant lesions of SpC-c-MYC mice express type II pneumocyte- and progenitor- cell markers. Paraffin embedded lung sections were stained as indicated. Clusters of pleomorphic cells that represent premalignant lesions in SpC-c-MYC mice were highlighted with red circles. Scale bar: 50 µm. (7.93 MB TIF) [file pone.0006029.s002.tif]

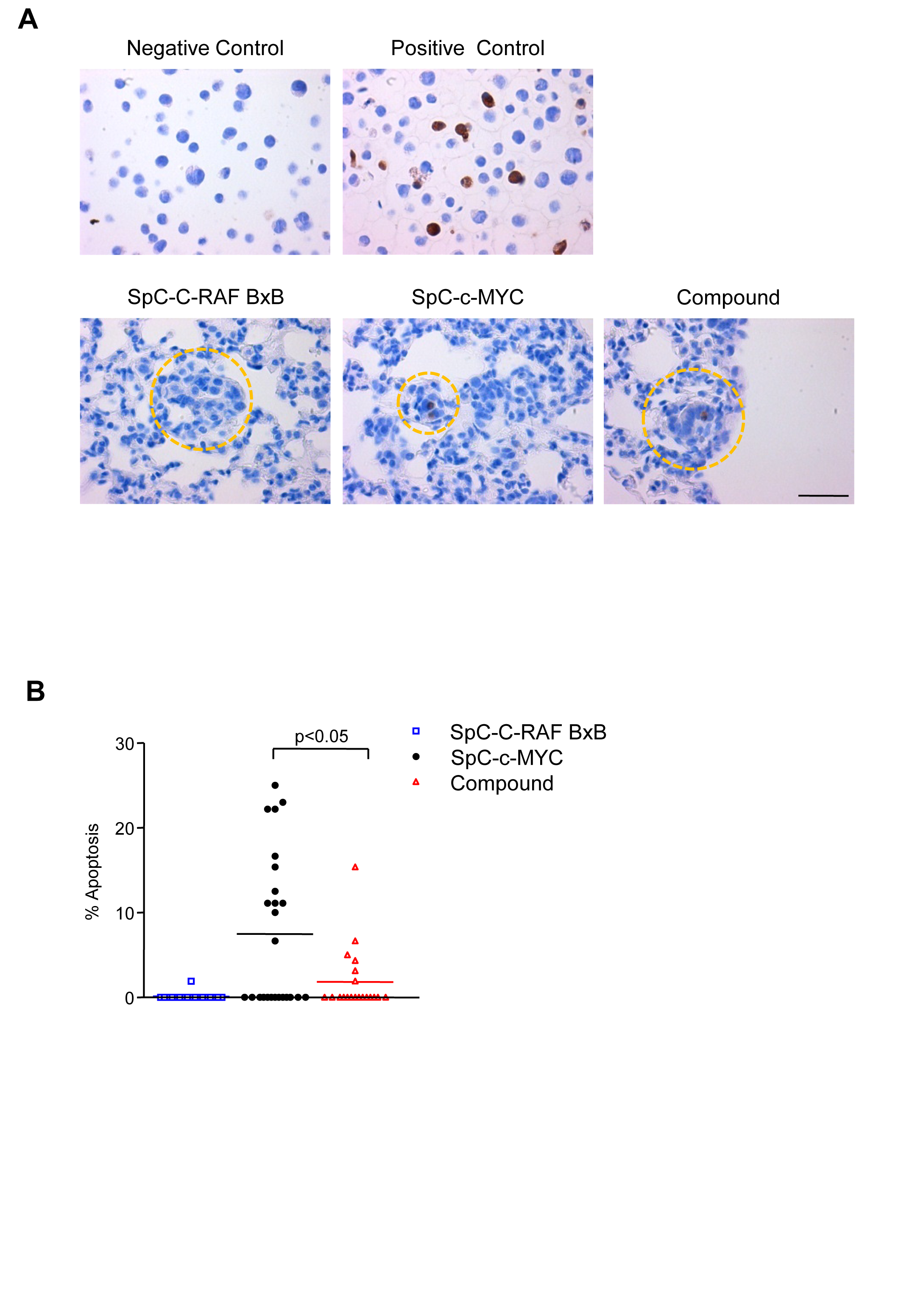

Supplement: Figure S2 — Rescue of cryptic MYC transformants by co-expression of RAF. (A) Paraffin embedded lung sections from all genotypes were stained for active caspase 3 (brown) to detect apoptotic cells. Yellow circles identify neoplastic lesions. Jurkat cells treated or untreated with etoposide were used as positive and negative control, respectively. Haematoxylin (Blue) was used for counterstaining. Scale bar: 50 µm. (B) Quantitation of apoptotic cells for indicated genotypes. At least 40 randomly selected lesions from 3 mice per genotype were analysed for quantitation of apoptotic cells. (2.28 MB TIF) [file pone.0006029.s003.tif]

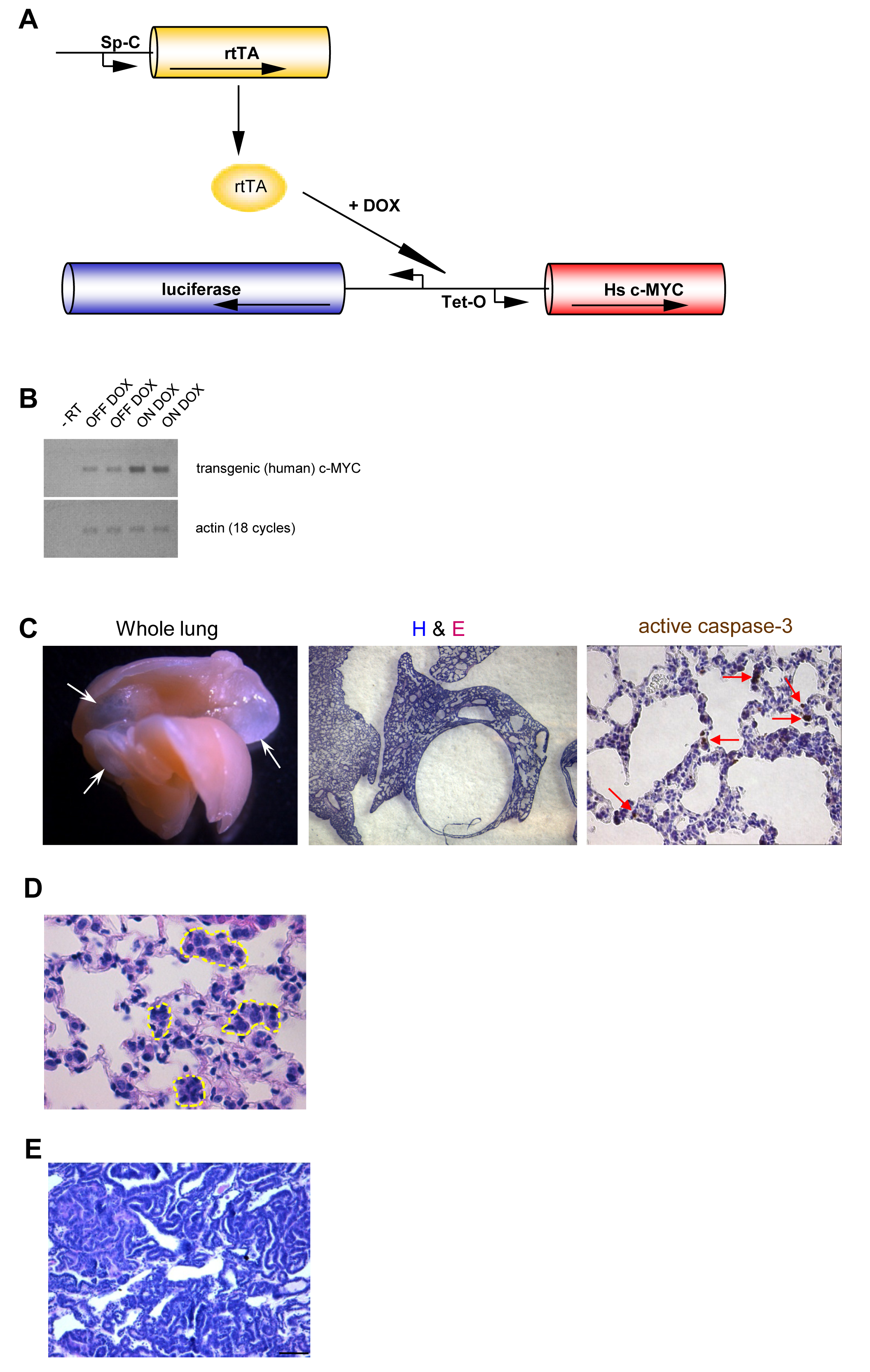

Supplement: Figure S3 — Conditional expression of c-MYC in lung alveolar type II cells induces tissue destruction. (A) Schematic diagram showing the generation of compound (SpC-rtTA/tet-O-c-MYC) mice conditionally expressing c-MYC in type II pneumocytes. Hs: Human. (B) Semi-quantitative RT-PCR showing inducible transgenic c-MYC (human) expression in lungs of compound mice after one week DOX administration. (C) Lung of a compound (SpC-rtTA/tet-O-c-MYC) mouse after four weeks induction shows severe tissue loss (white arrows) as evident from inspection of whole lung and the H&E stained section. Active caspase 3 staining identifies apoptotic cells (brown cells indicated by red arrows) in alveoli one day after doxycycline administration. Haematoxylin (blue) was used for counterstaining. (D) H&E staining of a lung section from four weeks-induced compound (SpC-rtTA/tet-O-c-MYC) mouse shows isolated pleomorphic cell clusters (yellow dash marking). (E) H&E staining of a lung section from 26 weeks-induced compound (SpC-rtTA/tet-O-c-MYC) mouse shows a lung adenocarcinoma with columnar cells. Scale bar: 100 µm. (3.53 MB TIF) [file pone.0006029.s004.tif]

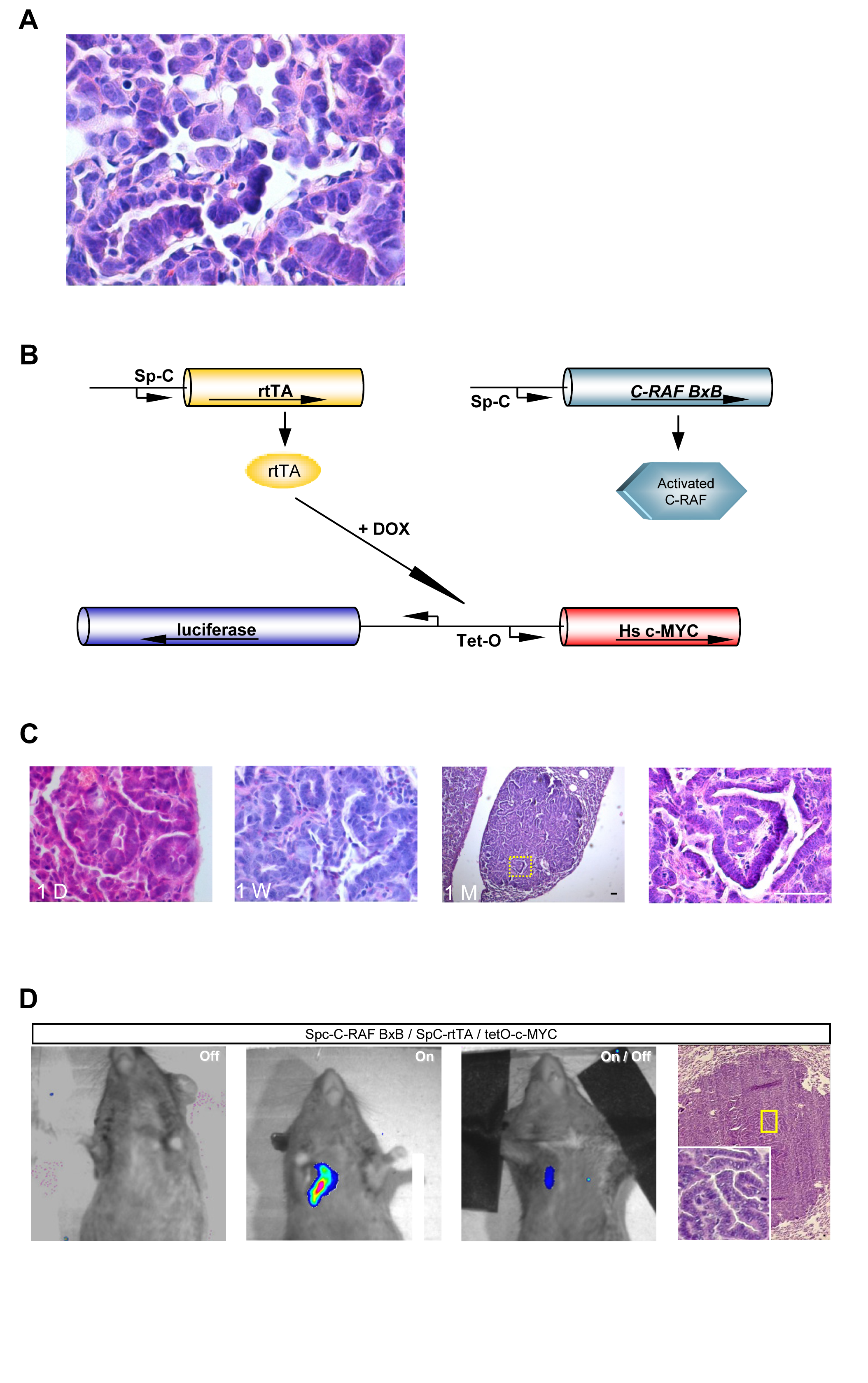

Supplement: Figure S4 — Induction of phenotypic switch from cuboidal to alveolar papillary/columnar epithelial cells (APECs). (A) H&E staining of a mixed (cuboidal and columnar) lung tumor section from four months old compound (SpC-C-RAF BxB/SpC-c-MYC) mouse. (B) Schematic diagram showing the generation of triple transgenic compound (SpC-C-RAF BxB/SpC-rtTA/tetO-c-MYC) mice. (C) H&E staining of lung tumor sections from inducible (SpC-C-RAF BxB/SpC-rtTA/tetO-c-MYC) compound mice shows the kinetics of columnar cell appearance. D: day, W: week, M: month. Right hand panel is a magnification of the yellow box. Scale bar: 100 µm. (D) Six weeks old compound mice were imaged for in vivo luciferase expression following one week On DOX/4 weeks Off DOX schedule demonstrating inducibility. H&E staining of a lung tumor section of the On/Off DOX mouse. Inset highlights persistent papillary tumor area indicated by yellow box. (3.75 MB TIF) [file pone.0006029.s005.tif]

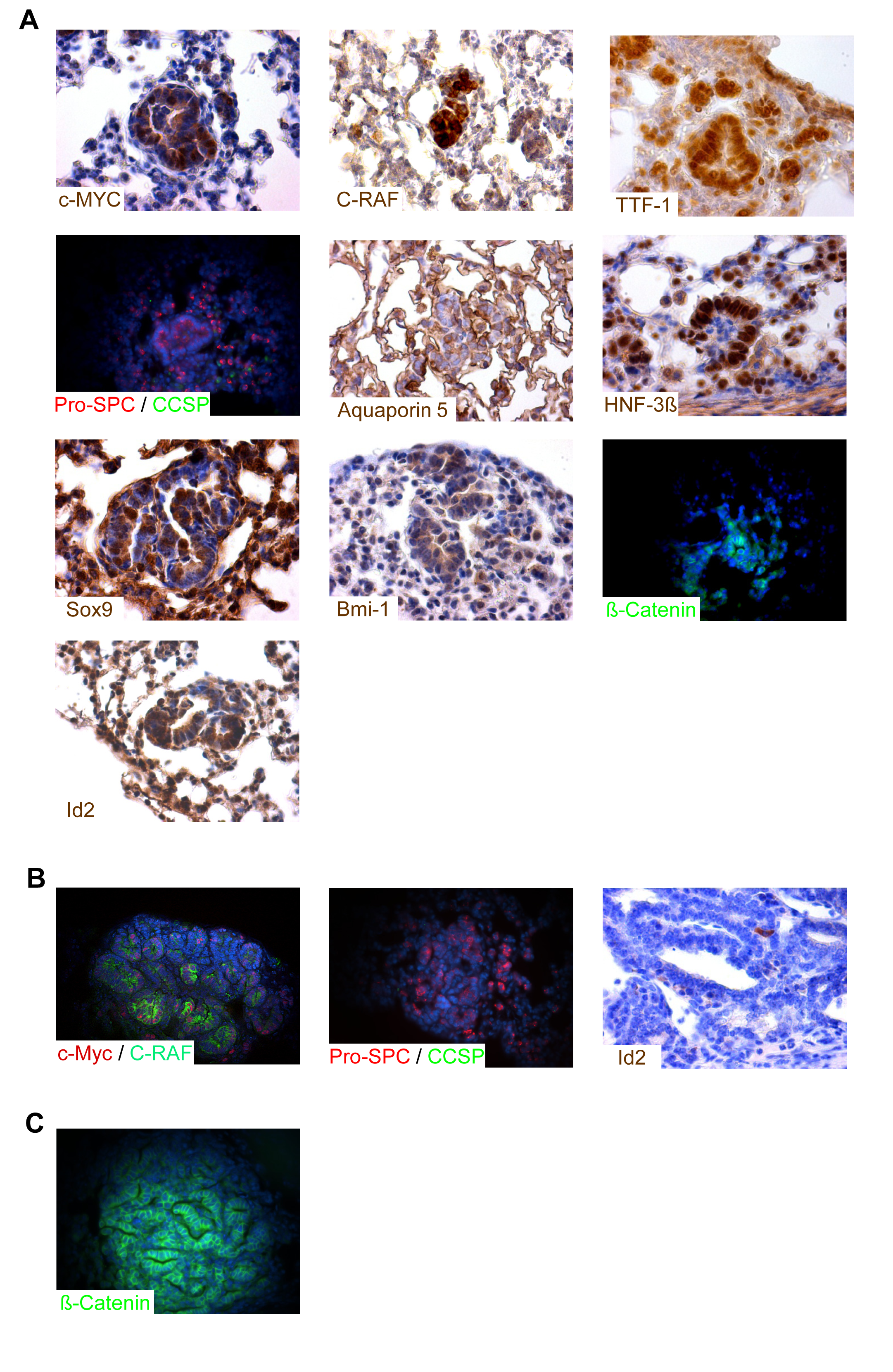

Supplement: Figure S5 — Immunostaining of emerging and late tumors of compound mice (SpC-C-RAF BxB/SpC-c-MYC) for lineage and progenitor cell markers. (A) Emerging tumors from 2 weeks-old mice were stained as indicated. (B) Late tumors from 10–16 months-old mice were stained as indicated. Markers are as indicated. In the case of fluorescence stainings colours correspond to the indicated proteins. Dapi (blue) illustrates nuclei. (C) β-catenin (green) staining of a lung tumor showing membrane localisation. Dapi (blue) illustrates nuclei. (7.15 MB TIF) [file pone.0006029.s006.tif]

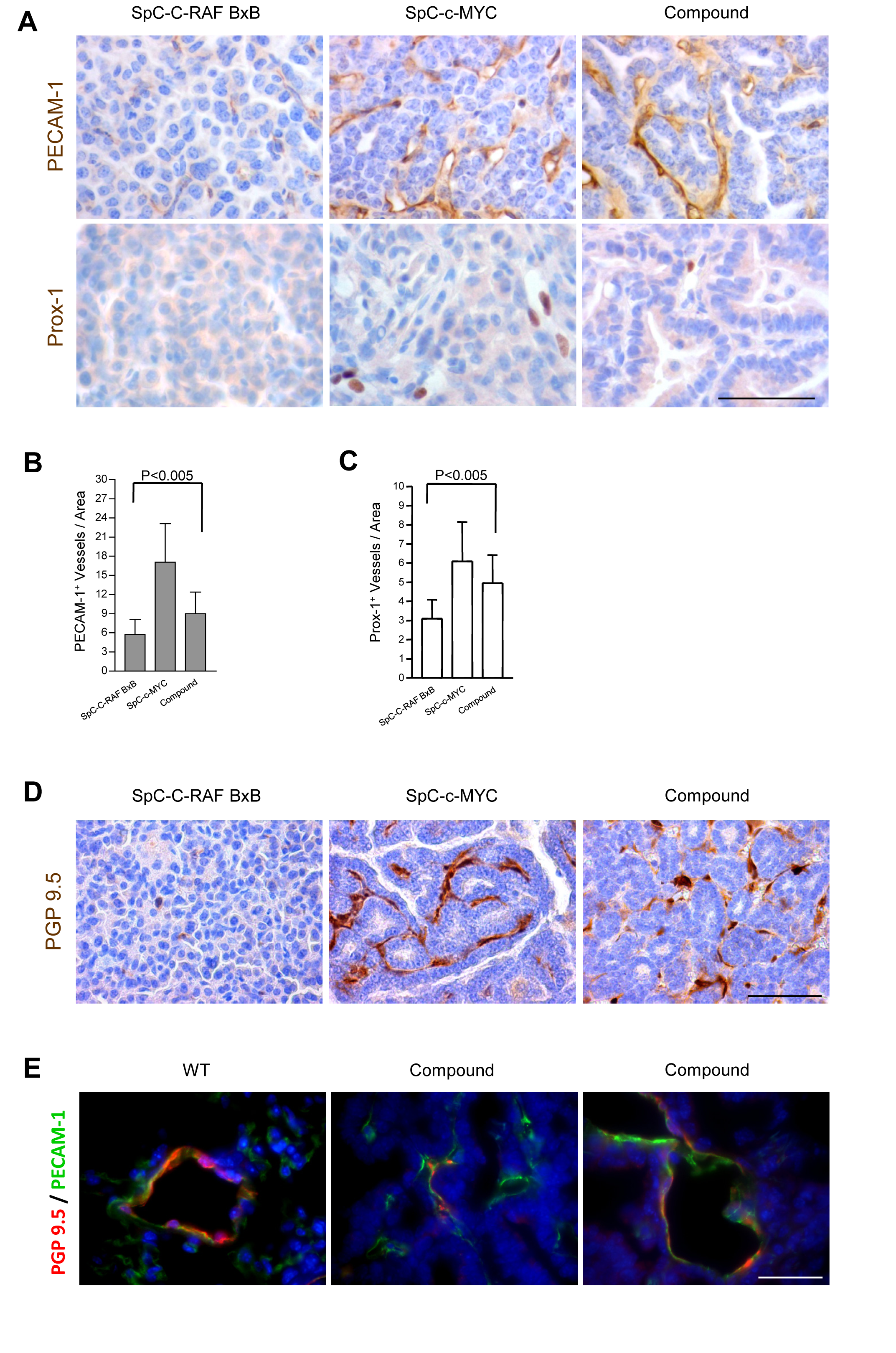

Supplement: Figure S6 — Induction of angiogenic switch by c-MYC. (A) Immunostaining of lung tumor sections from control and metastatic animals of indicated genotypes for blood (Pecam 1) and lymph (Prox 1) vessels. Scale bar 100 µm. (B, C) Quantitation of vessel density. 5 mice per genotype were analysed. Values represent SD of mean. P values are as indicated. (D) PGP 9.5 immunostaining of lung tumor sections. Scale bar 100 µm. (E) Frozen lung sections from wild type (wt) and compound mice were stained for PGP 9.5 (red) and PECAM-1 (green) for co-expression. Dapi illustrates nuclei. Scale bar 50 µm. (5.74 MB TIF) [file pone.0006029.s007.tif]

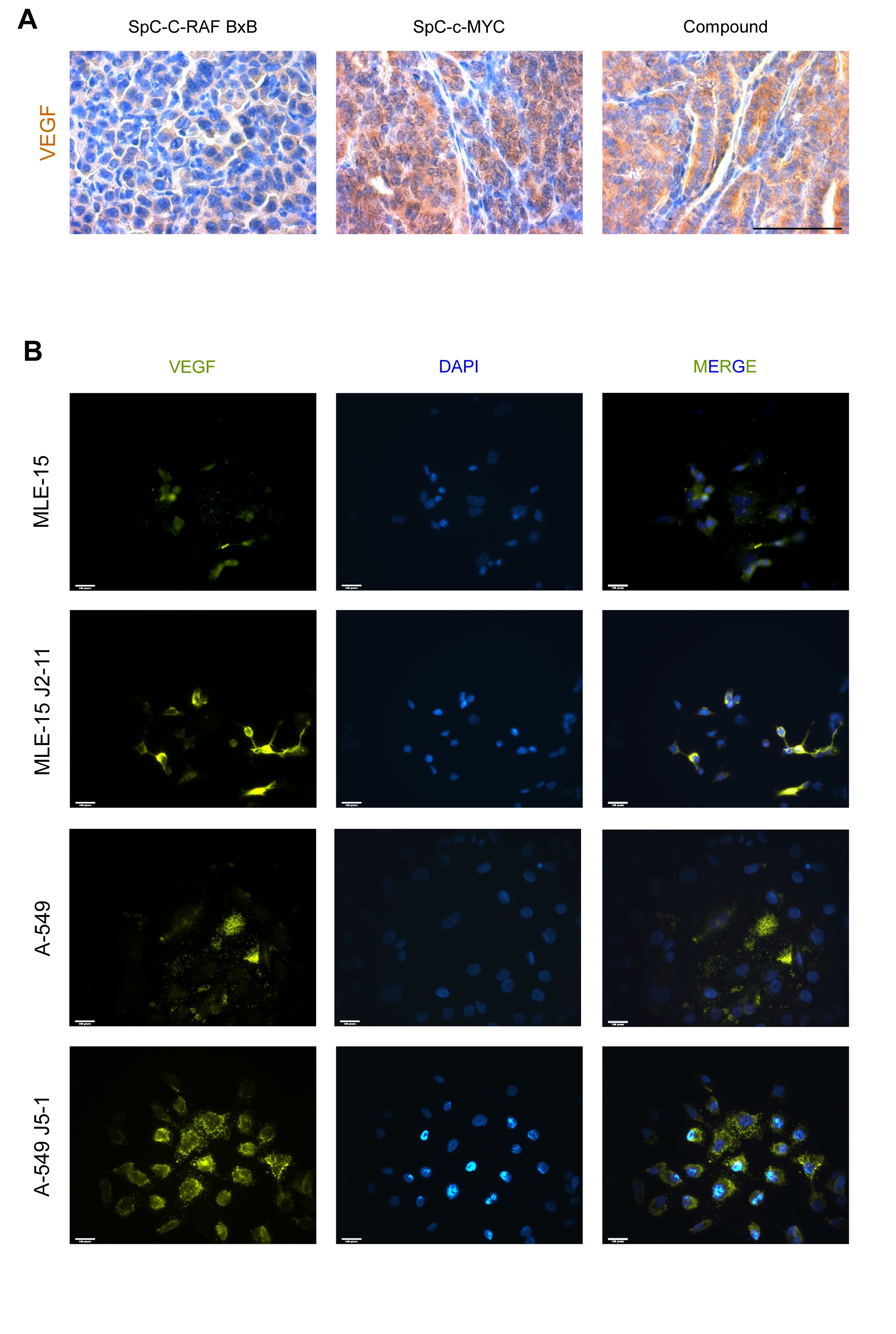

Supplement: Figure S7 — VEGF induction by MYC in primary lung tumors and NSCLC cell lines. (A) Immunostaining of lung tumor sections from age-matched mice of the indicated genotypes for VEGF (brown). Haematoxylin (blue) was used for counterstaining. Scale bar: 100 µm. (B) Immunocytochemistry of human A-549 and mouse MLE-15 NSCLC cell lines for VEGF (green). Note increased VEGF expression in cells infected with Myc expressing retroviruses J5-1 and J2-11. One representative cell clone is shown for each virus. Dapi (blue) illustrates nuclei. Scale bar: 10 µm. (3.77 MB TIF) [file pone.0006029.s008.tif]

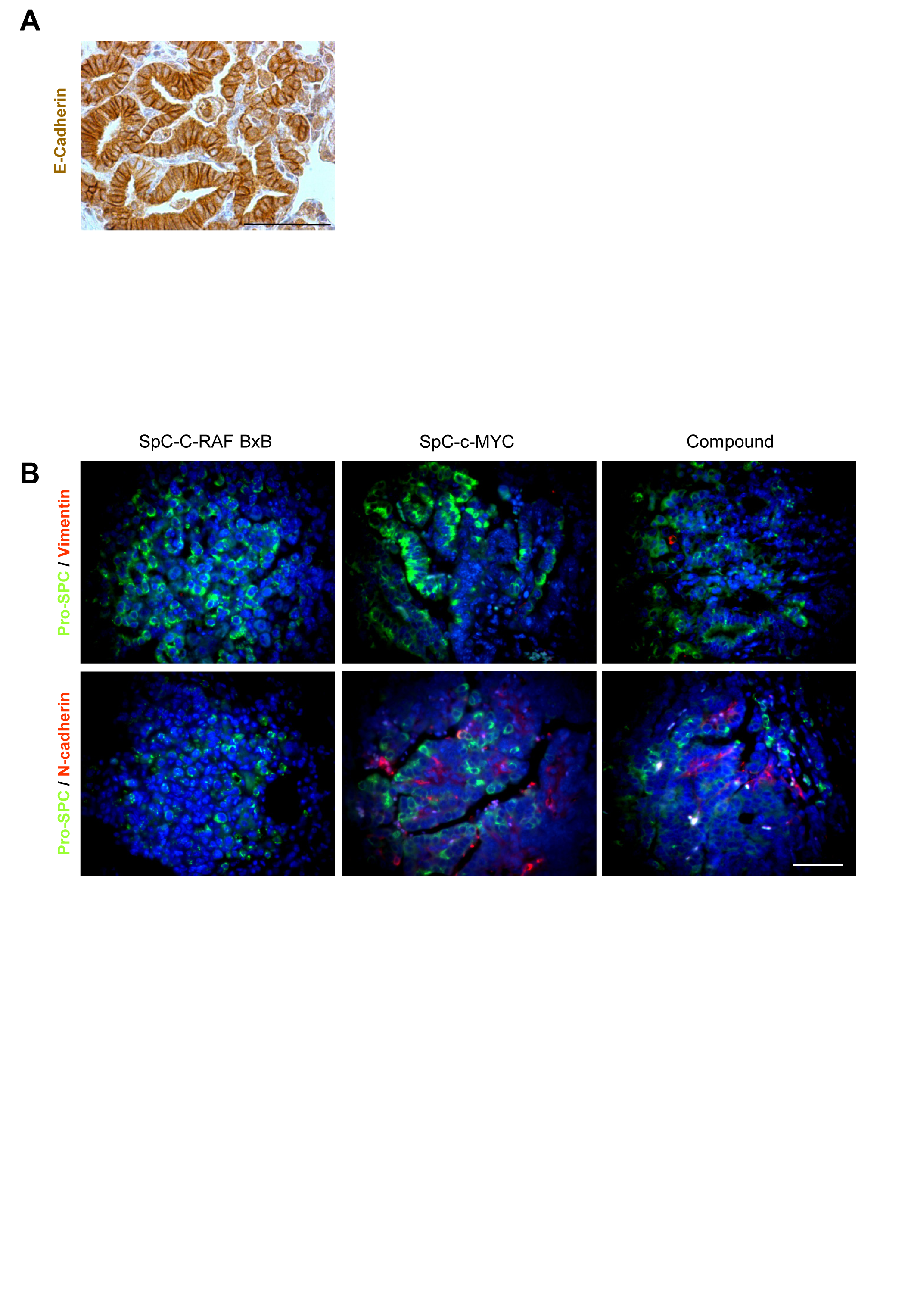

Supplement: Figure S8 — No evidence for EMT in tumor progression. (A) E-cadherin immunofluorescence staining (brown) of lung tumor section from a 12 months old compound mouse. Scale bar: 100 µm. (B) Paraffin embedded lung tumor sections from control and metastatic animals were stained for EMT markers as indicated. Tumor cells were marked by pro SP-C (green) staining. Dapi (blue) illustrates nuclei. Scale bar: 100 µm. (3.53 MB TIF) [file pone.0006029.s009.tif]

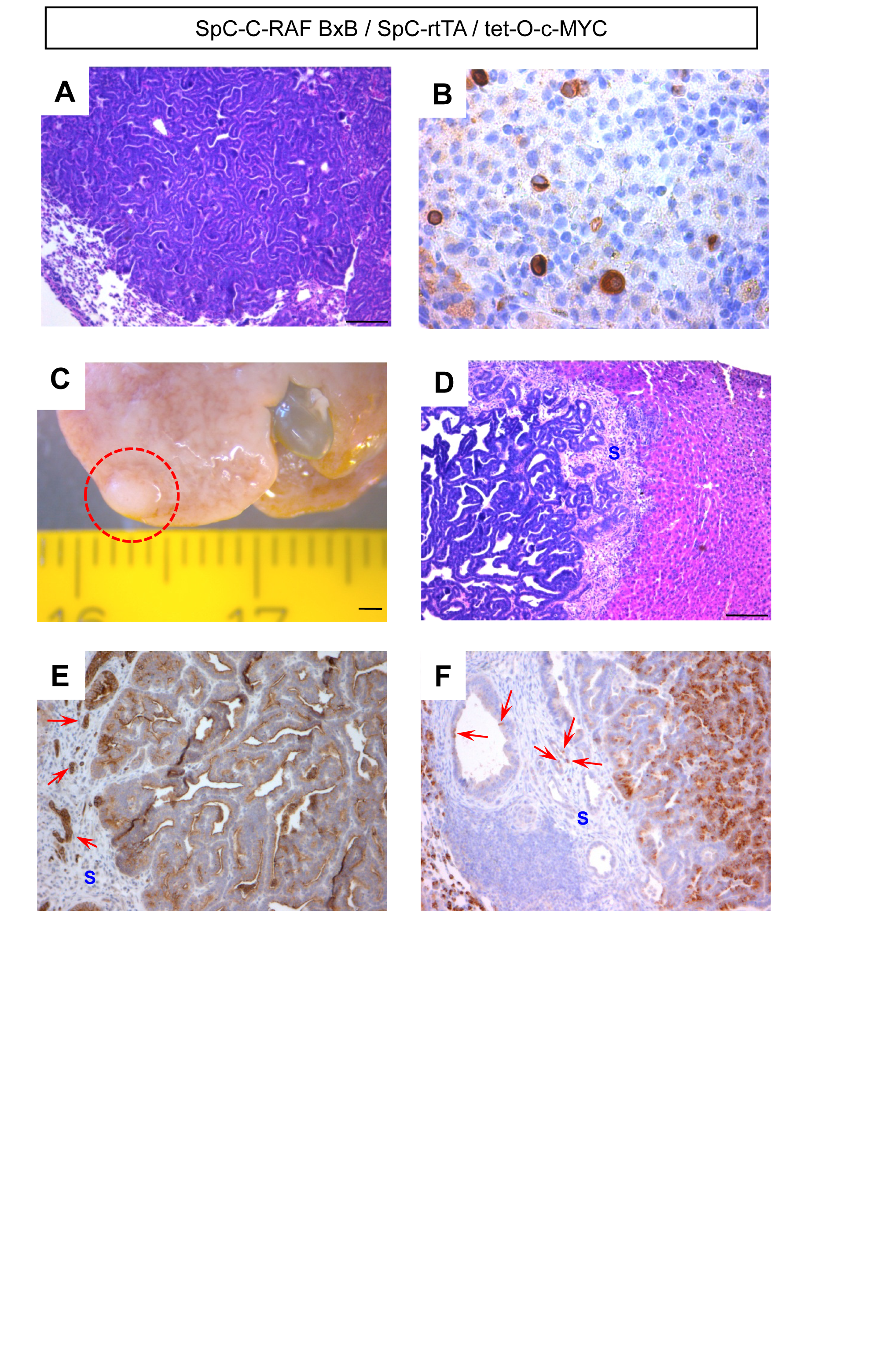

Supplement: Figure S9 — Inducible expression of c-MYC in SpC-C-RAF BxB lung tumors give rise to liver metastasis with APECs. (A) H&E staining of a lung section from a seven months induced compound mouse shows large lung tumors with columnar cell. Scale bar: 100 µm. (B) Pan-cytokeratin staining (brown) of a regional lymph node section from eleven months-induced compound mouse (SpC-C-RAF BxB/SpC-rtTA/tet-O-c-MYC) shows micrometastasis. (C) Inspection of a liver from a seven months induced compound (SpC-C-RAF BxB/SpC-rtTA/tet-O-c-MYC) mouse shows a tumor nodule in the liver (red circle). (D) H&E staining of the liver metastasis shown in C demonstrates papillary tumors with stroma (S). Scale bar: 100 µm. (E, F) Pan-cytokeratin (E) and pro SP-C immunostaining of the liver metastasis shown in C. Isolated pan-cytokeratin and pro SP-C positive tumor cells that were embedded in the stroma (S) were indicated by red arrows. (6.17 MB TIF) [file pone.0006029.s010.tif]

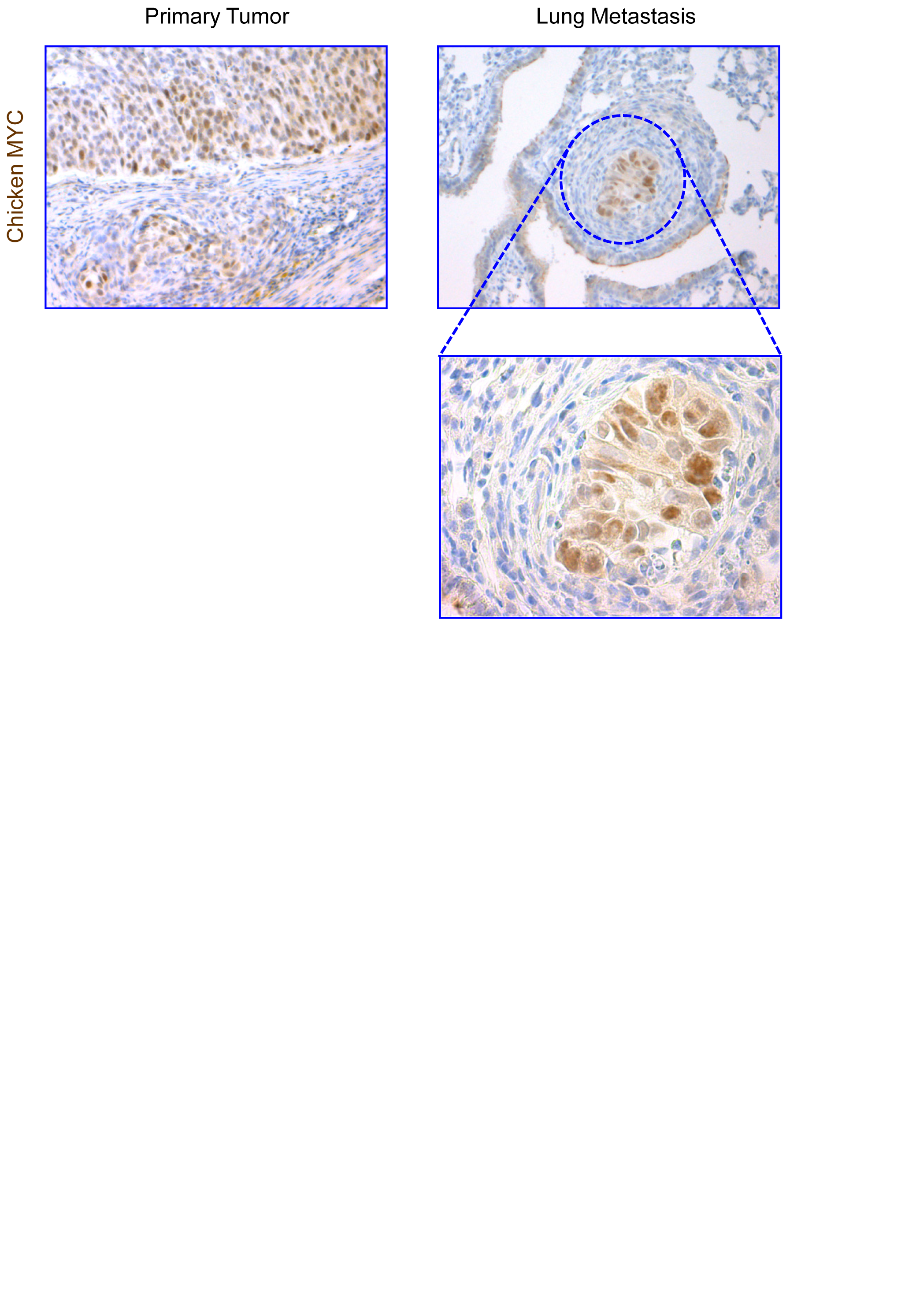

Supplement: Figure S10 — Staining of primary tumor and lung metastasis developing after subcutenous injection of A-549 J5-1 cells for chicken c-MYC. (3.35 MB TIF) [file pone.0006029.s011.tif]

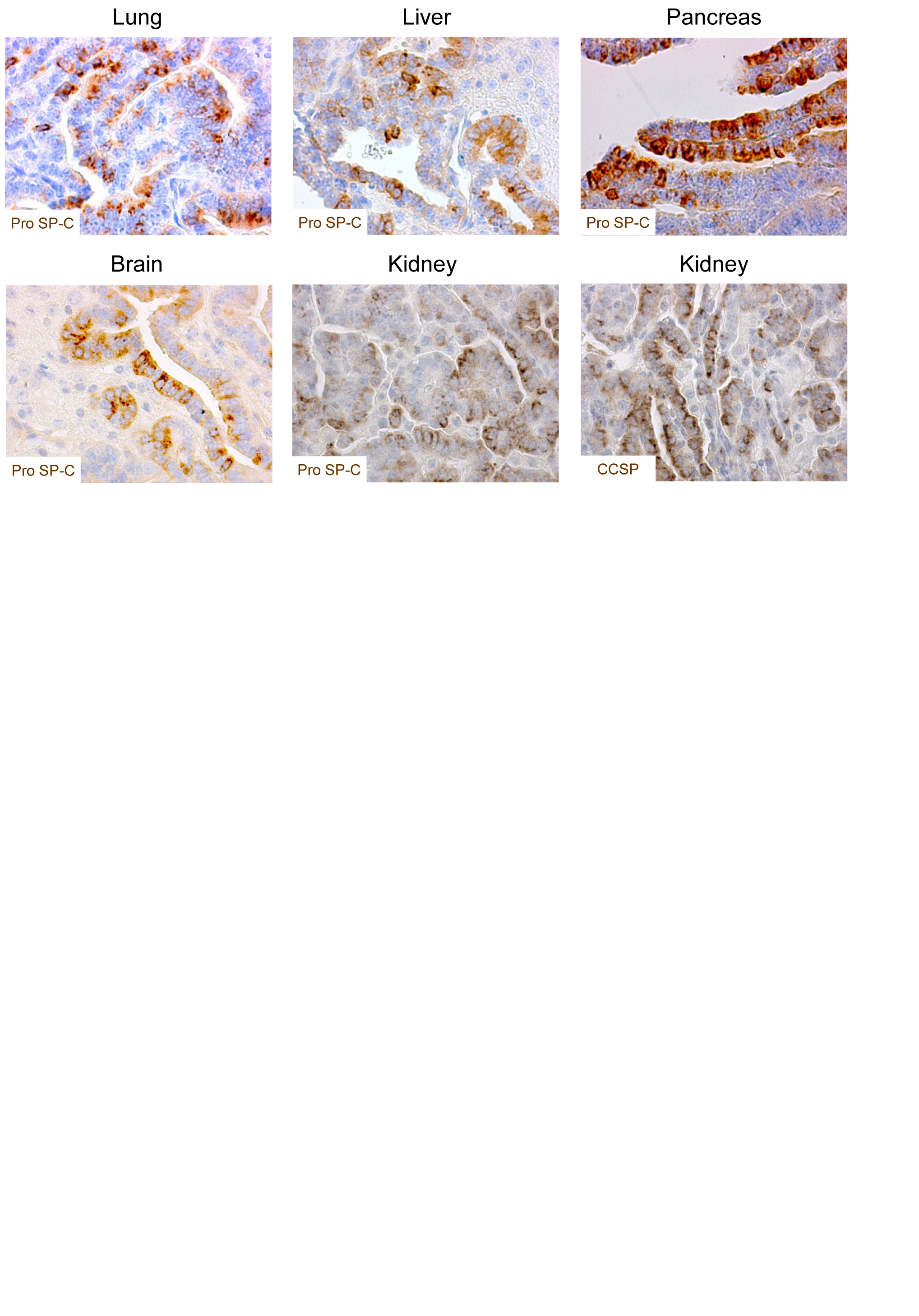

Supplement: Figure S11 — Histopathology of metastasis to distant organs in old SpC-c-MYC mice. Multiple target organs are involved in case of a mutant K-Ras positive SpC-c-MYC lung tumor at age 20,5 months. Tissue sections were stained with indicated markers which identifies derivation of metastasis from lung adenocarcinoma. Haematoxylin (blue) was used for counterstaining. (3.71 MB TIF) [file pone.0006029.s012.tif]

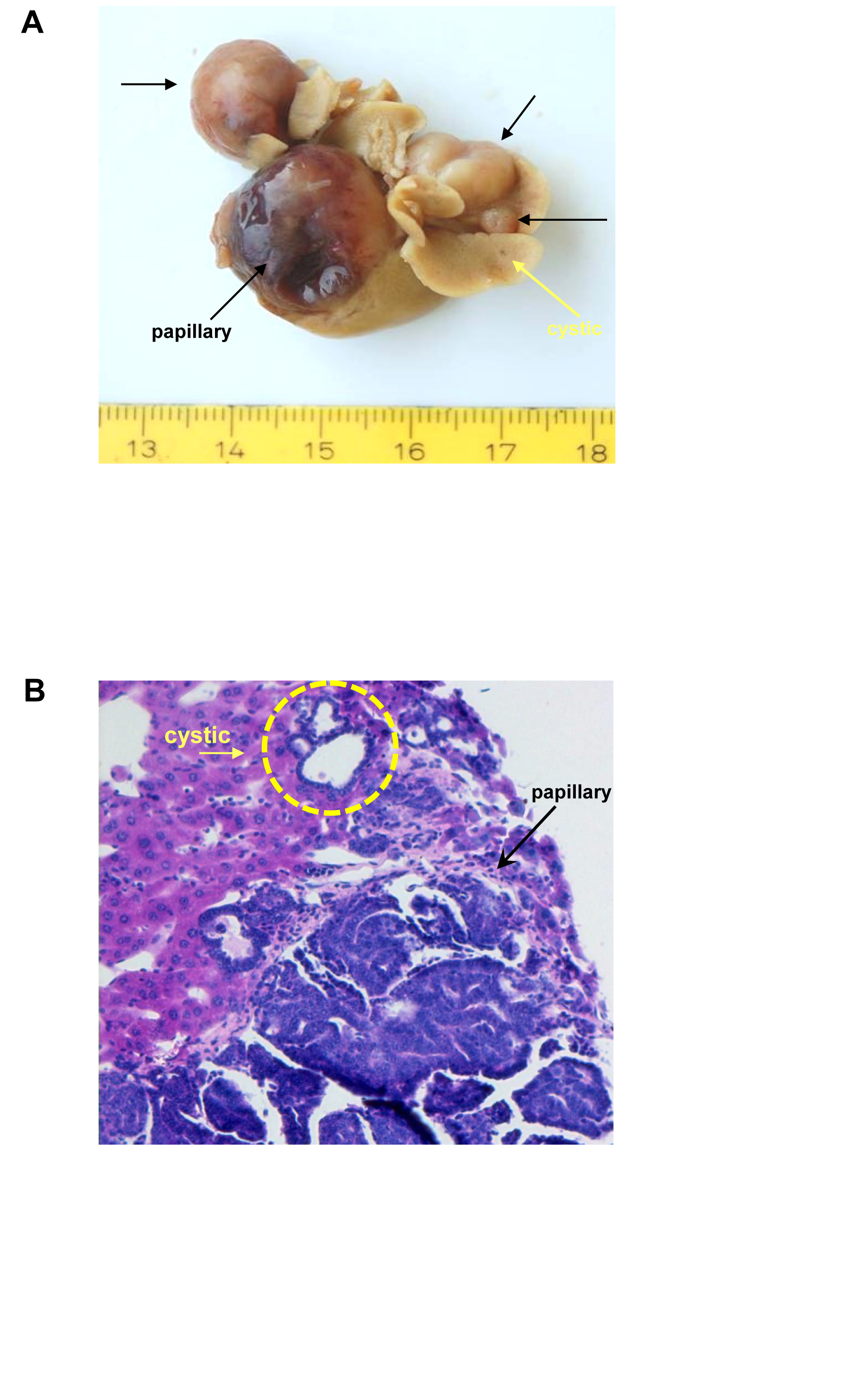

Supplement: Figure S12 — Cystic and papillary forms coexist in late liver metastasis. (A) Macroscopic liver metastasis in an 18 months old SpC-c-MYC mouse. Note multiple solid tumor nodules (papillary, black arrows) and cystic lesion (yellow arrow). (B) H&E staining of liver metastasis confirm coexistence of both papillary and cystic (yellow circle) lesions in the same organ. (2.83 MB TIF) [file pone.0006029.s013.tif]

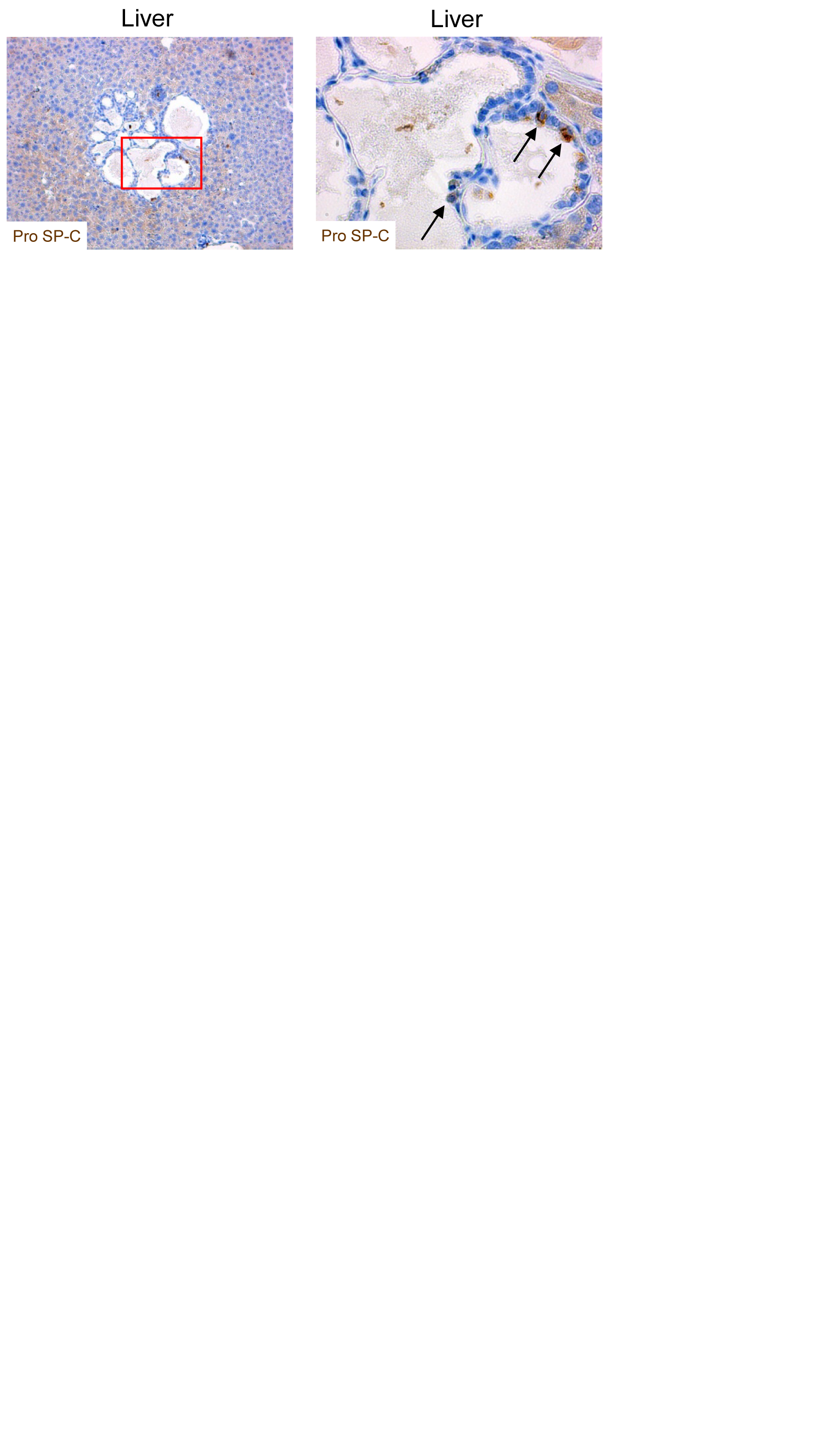

Supplement: Figure S13 — Cystic liver metastasis of a 13 months old SpC-c-MYC mouse. High magnification of red inset illustrates presence of pro SP-C positive (brown) cells (arrows) in the epithelial layer lining the cyst. Haematoxylin (blue) was used for counterstaining. (1.35 MB TIF) [file pone.0006029.s014.tif]

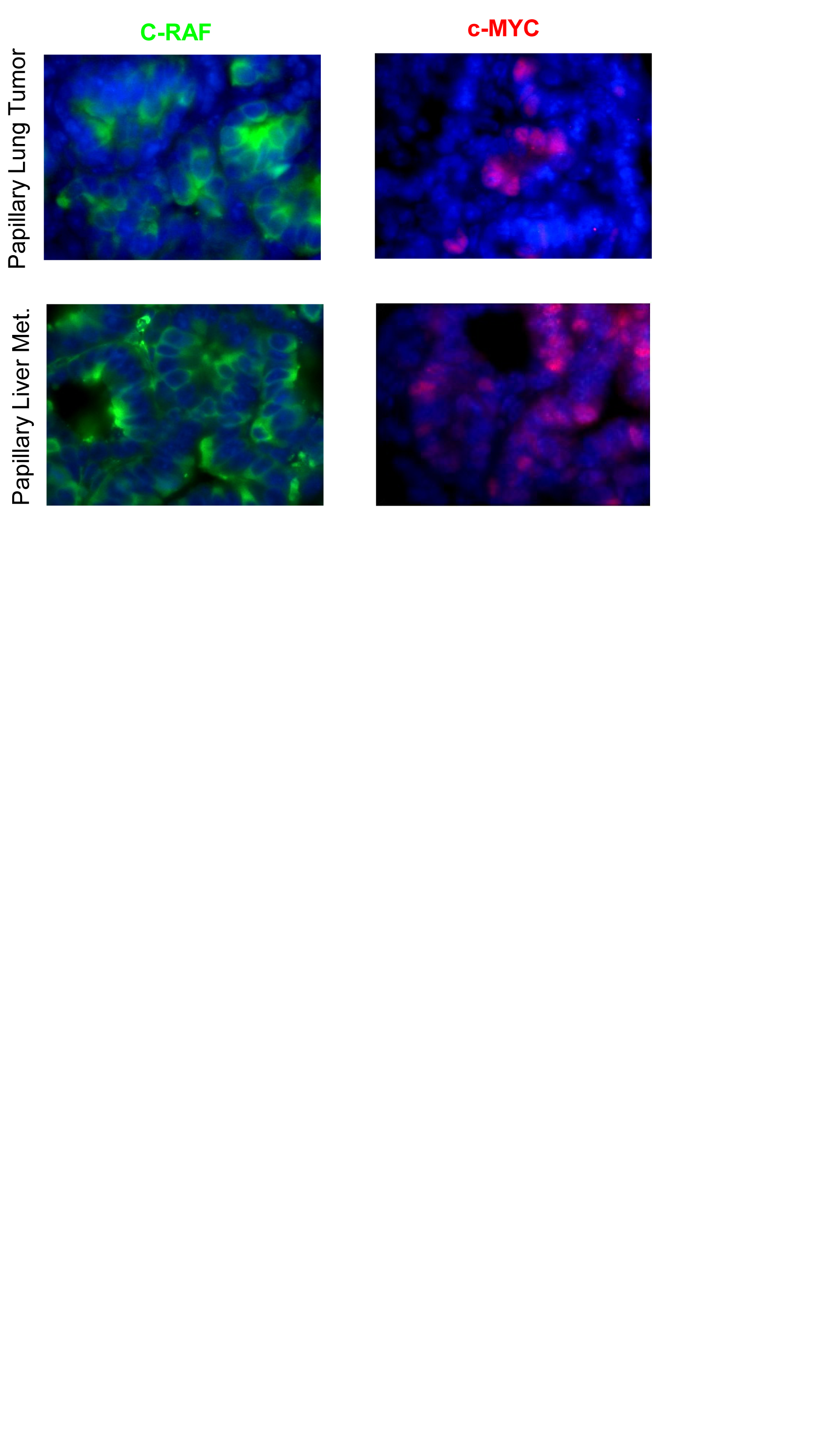

Supplement: Figure S14 — Expression of transgene markers in liver metastases. Immunofluorescence staining of papillary lung tumors and liver metastasis for expression of C-RAF (green) and c-MYC (red) transgenes. (1.36 MB TIF) [file pone.0006029.s015.tif]

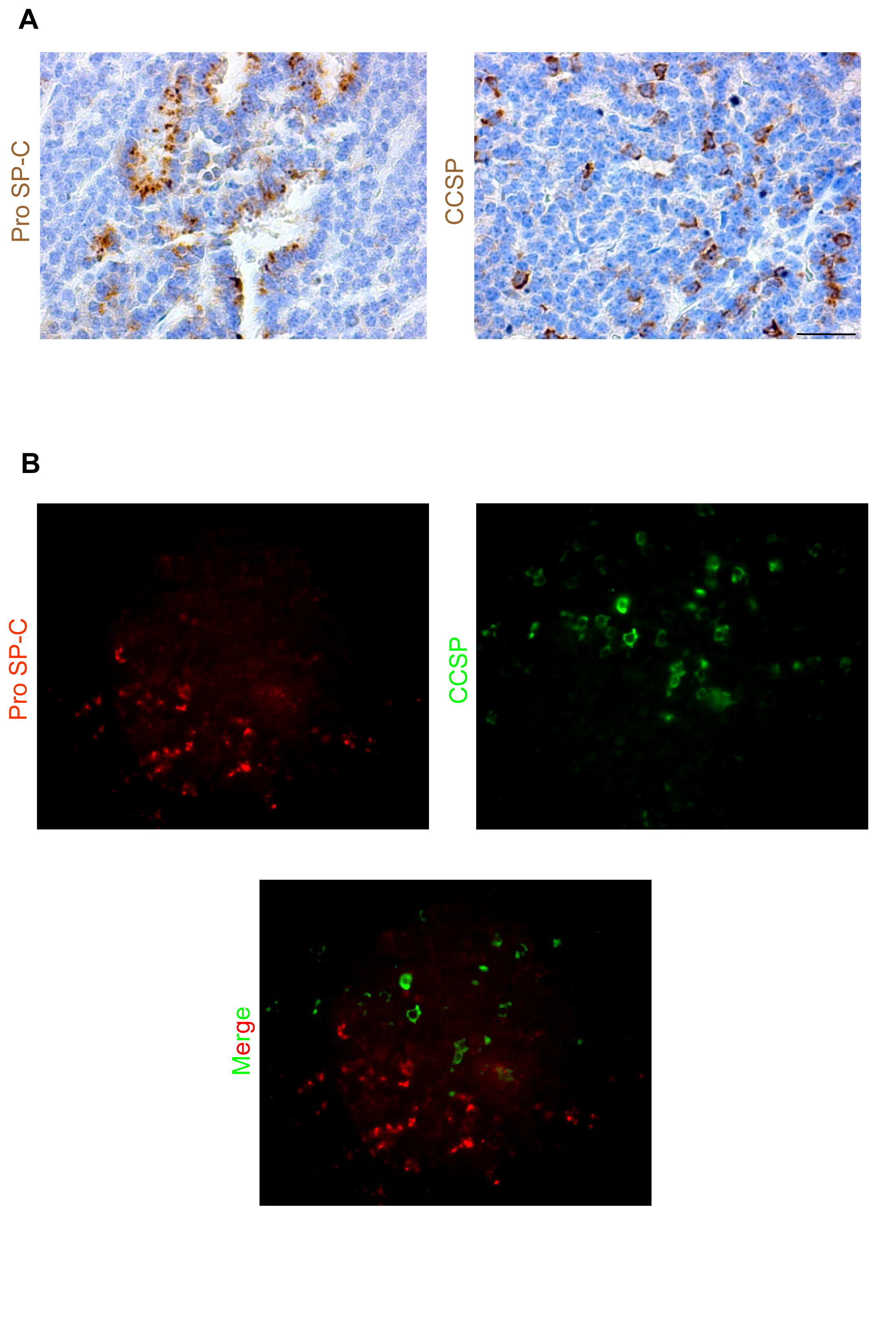

Supplement: Figure S15 — No evidence for BASC in liver metastasis. (A) Paraffin embedded sections from 18 months liver metastasis were stained for CCSP and pro SP-C. Haematoxylin was used for counterstaining. Scale bar: 50 µm. (B) The same sections were subsequently examined for co-expression of CCSP (green) and pro SP-C (red) to search for BASCs. Dapi (blue) illustrates nuclei. (3.65 MB TIF) [file pone.0006029.s016.tif]

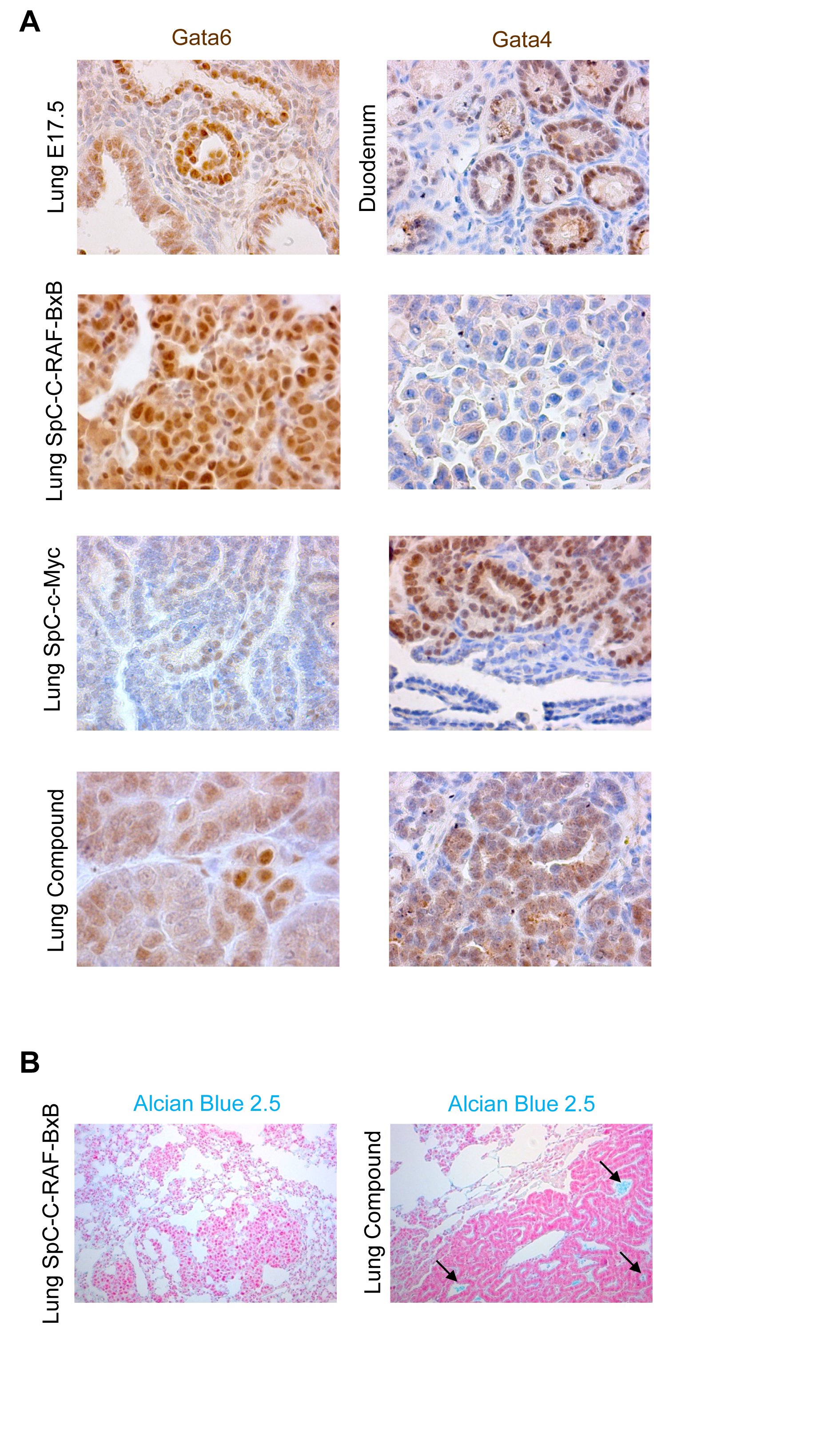

Supplement: Figure S16 — Gata6/Gata4 switch in SpC-c-MYC and compound mice. (A) Reciprocal expression of Gata4 and Gata6 in primary tumor of SpC-c-MYC and compound mice. Stainings as indicated. Note decrease in the level of Gata6 expression concomitant with heterogenous Gata4 expression in late stage lung tumors. Duodenum and embryonic lung sections were used for positive control for Gata4 and Gata6, respectively. (B) Alcian Blue 2.5 expression in lung tumors from indicated genotypes. Sections from 12 months old mice were stained with Alcian Blue for mucin secretion (blue stains, indicated by arrows). (6.04 MB TIF) [file pone.0006029.s017.tif]

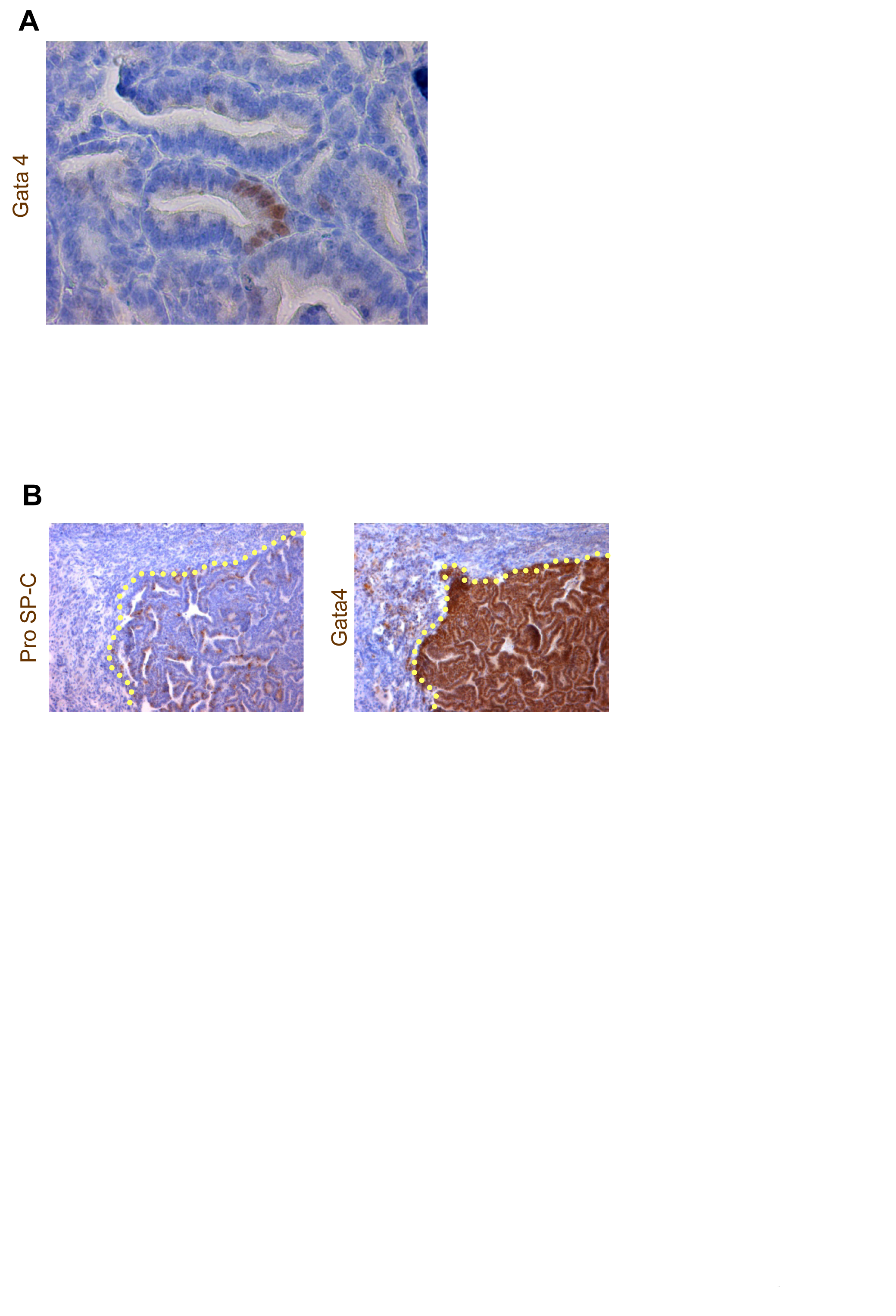

Supplement: Figure S17 — Early and mutually exclusive expression of ectopic Gata4. (A) A representative lung section from a six-months-old compound (SpC-C-RAF BxB/SpC-c-MYC) mouse with an array of Gata4 expressing (brown) cells. (B) Immunohistochemistry for Gata4 and pro SP-C in serial sections of a lung tumor from a 18-monthd-old SpC-c-MYC single transgenic mouse illustrating mutually exclusive expression of both markers. (2.63 MB TIF) [file pone.0006029.s018.tif]
